# A distance-type measure approach to the analysis of copy number variation in DNA sequencing data

**DOI:** 10.1186/s12864-019-5491-x

**Published:** 2019-04-04

**Authors:** Bipasa Biswas, Yinglei Lai

**Affiliations:** 1 Diagnostics Devices Branch 1, FDA/CDRH/OSB-DBS, White Oak Bldg #66, Room 2222, 10903 New Hampshire Avenue, Silver Spring, MD 20993 USA; 2 Department of Statistics and Biostatistics Center, The George Washington University, Rome Hall, 7th Floor, 801, 22nd Street NW, Washington D.C, 20052 USA

**Keywords:** Genome-wide sequencing, DNA, Copy number variation, Distance-type measure, Geometric distribution, Mixture model

## Abstract

**Background:**

The next generation sequencing technology allows us to obtain a large amount of short DNA sequence (DNA-seq) reads at a genome-wide level. DNA-seq data have been increasingly collected during the recent years. Count-type data analysis is a widely used approach for DNA-seq data. However, the related data pre-processing is based on the moving window method, in which a window size need to be defined in order to obtain count-type data. Furthermore, useful information can be reduced after data pre-processing for count-type data.

**Results:**

In this study, we propose to analyze DNA-seq data based on the related distance-type measure. Distances are measured in base pairs (bps) between two adjacent alignments of short reads mapped to a reference genome. Our experimental data based simulation study confirms the advantages of distance-type measure approach in both detection power and detection accuracy. Furthermore, we propose artificial censoring for the distance data so that distances larger than a given value are considered potential outliers. Our purpose is to simplify the pre-processing of DNA-seq data. Statistically, we consider a mixture of right censored geometric distributions to model the distance data. Additionally, to reduce the GC-content bias, we extend the mixture model to a mixture of generalized linear models (GLMs). The estimation of model can be achieved by the Newton-Raphson algorithm as well as the Expectation-Maximization (E-M) algorithm. We have conducted simulations to evaluate the performance of our approach. Based on the rank based inverse normal transformation of distance data, we can obtain the related *z*-values for a follow-up analysis. For an illustration, an application to the DNA-seq data from a pair of normal and tumor cell lines is presented with a change-point analysis of *z*-values to detect DNA copy number alterations.

**Conclusion:**

Our distance-type measure approach is novel. It does not require either a fixed or a sliding window procedure for generating count-type data. Its advantages have been demonstrated by our simulation studies and its practical usefulness has been illustrated by an experimental data application.

**Electronic supplementary material:**

The online version of this article (10.1186/s12864-019-5491-x) contains supplementary material, which is available to authorized users.

## Background

The next generation sequencing technology has advanced significantly during the recent decade. It allows us to obtain a large amount of short DNA sequence (DNA-seq) reads at a genome-wide level [1]. DNA-seq data have been increasingly collected during the recent years [2, 3]. Count-type data analysis is a widely used approach for DNA-seq data [4]. However, for data pre-processing, a moving window with the related window size need to be defined in order to obtain count-type data [5]. During the data pre-processing, given a window of genomic region, the observations within the region are summarized. However, useful information from the original data can be reduced after data pre-processing for count-type data.

DNA-seq has been widely used for the detection of copy number variation/alteration [1, 4]. Both copy number variation (CNV) analysis and copy number alteration (CNA) analysis are based on the assumption that the observed number of short reads of a local genomic region is proportional to the underlying copy number of DNA. CNV/CAN can also be discovered by other biomedical techniques such as fluorescent in situ hybridization, comparative genomic hybridization, array comparative genomic hybridization, and by virtual karyotyping with SNP arrays [2, 6, 7]. The next generation sequencing technology allows us to obtain millions of short DNA reads in a relatively short amount of time [8–10]. Digital karyotyping is a simple and powerful method [6]. Many statistical methods for CNV/CAN analysis are based on the count-type DNA-seq data [2, 3, 5, 11]. It is also necessary to consider GC content bias in the DNA-seq data [12]. The GC content can be calculated based on the Guanine (G) and Cytosine (C) bases in a reference genome. GC content bias refers to the dependence between the sequencing data and the related GC content.

Due to the limitations of current sequencing techniques, the sequencing data cannot be obtained for certain genomic regions. Then, the related count-type measure is simply zero. For a DNA sequence containing such a genomic region, a large gap without any short reads is observed. Then, the related distance-type measure can be very large (considered outliers statistically, see below for the definition of distance-type measure) and an artificial censoring can be considered.

In this study, we consider distance-type measure. Distances are measured in base pairs (bps) between two adjacent alignments of short reads mapped to a reference genome. Our distance-type measure approach is novel. It does not require either a fixed or a sliding window procedure for generating count-type data. Furthermore, we propose artificial censoring for the distance data so that distances larger than a given value are considered potential outliers. Our purpose is to simplify the pre-processing of DNA-seq data.

Statistically, we consider a mixture of right censored geometric distributions to model the distance data. Additionally, to reduce the GC-content bias, we extend the mixture model to a mixture of generalized linear models (GLMs). Our approach can be considered as an alternative to the method proposed by Shen and Zhang [11]. The estimation of model can be achieved by on the Newton-Raphson algorithm as well as the Expectation-Maximization (E-M) algorithm. Then, based on the rank based inverse normal transformation of distance data, we can obtain the related z-values for a follow-up analysis.

In the following, we first demonstrate how to conduct experimental data based simulation study to confirm the advantages of distance-type measure approach in both detection power and detection accuracy. Then, we introduce our statistical models and the related estimation procedure. The model performance can be demonstrated by simulation studies. The practical usefulness of our approach can be illustrated by an application to the DNA-seq data from a pair of normal and tumor cell lines [1] as well as a follow-up change-point analysis of z-values for the detection of DNA copy number alterations.

## Methods

**Fig. 1:**
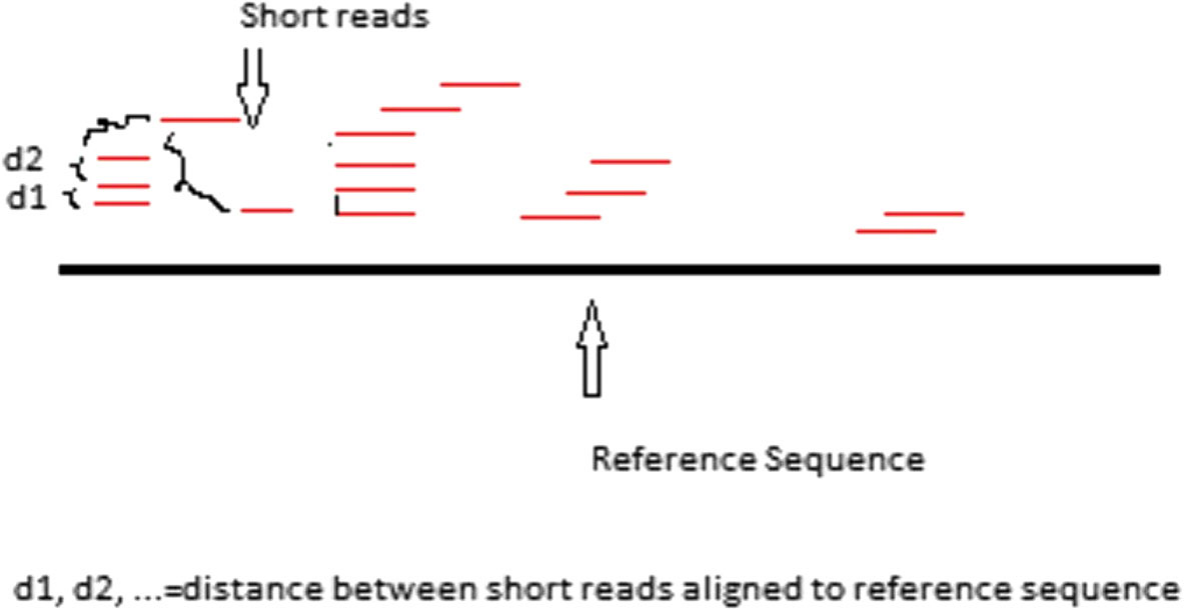
Illustration of distance measure, which is measured in base pairs (bps) between the first aligned base pair of two adjacent alignments of short reads mapped to a reference sequence

We consider a novel distance-type measure approach to the analysis of DNA-seq data. Distances are measured in base pairs (bps) between two adjacent alignments of short reads mapped to a reference genome. An illustration is shown in Fig. 1. A clear advantage is that the moving window method is no longer required for data pre-processing.

We first introduce how to compare the distance-type vs. count-type data so that more advantages of distance-type data can be confirmed. Then, we introduce a finite mixture [13, 14] of generalized linear models (GLMs) for modeling the distance between two adjacent reads with the adjustment to the GC-content. Artificial censoring of distance-type data can be considered so that potential outliers can be accommodated in the model. The basic distribution component for our method is the right censored geometric distribution. In the following, we briefly describe our method and the related estimation procedure. Their details are given in the Additional file 1.

### Comparing distance-type vs. count-type data

The widely used coverage depth (or count-type) based approach can be briefly described as follows. For a genomic region, it is divided into non-overlapping smaller regions (windows or bins) and the number of sequencing reads is counted for each bin. For example, if a genomic region is divided into 100 bins, then 100 count-type data can be obtained from these bins. The original sequencing read data are then simplified to count-type data by this approach. (Our proposed distance based approach is to maintain the overall structure as well as the overall size of original sequencing read data.)

In this study, the DNA sequencing data were collected for the detection of copy number variations. A certain type of data (distance or count) is generated from the original sequencing read data (by the distance based approach or the coverage depth based approach, respectively). Between these two types of data, our choice is decided based on the statistical power for detecting changes. To demonstrate the advantage of our approach, we perform a simulation study to evaluate whether the distance based approach can be more statistically powerful than the coverage depth based approach. We consider a relatively simple scenario: if two genomic regions are known to be different in copy numbers, then which approach is more likely to detect the change? Furthermore, can accurate detection of change location be achieved when the distance-type data are used in the copy number variation/alteration analysis?

To address which type of data is more likely to detect the change (detection power), we consider the following simulation. Our simulation data were generated based on some real experimental sequencing data (instead of simulating data completely from some statistical distributions). We generated data based on the region with position 10-30Mbps from the normal sample (as presented in the section *Application to Sequencing Data*, but we used the original sequencing read data). In this region, we randomly selected two bins with length L for each bin. For each of the original sequencing reads from the second bin, we removed it randomly with 50% probability. (The purpose was to simulate a deletion of 50% copy number.) We kept all the original sequencing reads from the first bin. In this way, we simulated sequencing data for two genomic regions with different copy numbers. The distance-type data can be obtained as described at the beginning of Methods section. To obtain count-type data, we divided each region into bins with length W for each bin (and then counted the number of sequencing reads in each bin). To achieve an unbiased comparison between the distance-type data and the count-type data, we used the non-parametric Komogorov-Smirnov two-sample test to compare the empirical distributions of simulated data for two genomic regions. Notice that either the distance based approach or the coverage depth based approach is to generate a certain type of data (distance-type or count-type, respectively). It is necessary to compare which type of data can be more statistically powerful in the detection of changes. Our evaluation strategy does not depend on data type, as it does not require any parametric or statistical distribution assumptions.

We considered different values for L: 50,000, 200,000 and 1,000,000 bps for different lengths of copy number variations, as well as different values for W: 5000, 10,000 and 25,000 bps for different bin sizes. (We considered the range of bin size 5-25 kbps to avoid zero counts or very few count data.) For each setting, we repeated our simulation and analysis for 5000 times.

To address whether accurate detection of change location can be achieved (detection accuracy), we obtained more analysis results from the above simulation setting. In each simulation repetition, we considered the data for two genomic regions were connected. We evaluated how accurate the boundary (change) between two regions could be detected. As the data for two regions were ordered, the change location could be between any two consecutive data (for both distance-type and count-type). Therefore, we screened all the possible locations and considered the location with the lowest *p*-value as the identified change location. In this way, from each simulation repetition, we obtained the identified change location and its related p-value. Although the lowest p-value was considered, some cutoff values would still be usually considered in practice. As all the possible change locations were screened, a cutoff value lower than the conventional cutoff value 0.05 would be usually considered. Therefore, we considered the following cutoff values for the lowest *p*-values: 0.01, 0.001, 0.0001, 0.00001, 0.000001 and 0.0000001. Based on the results for detection power comparison (also see Table 1), the advantage of distance-type data was clear for short L and the advantage of count-type data became clear for long L. Therefore, we considered several moderate L values: 150,000, 300,000 and 450,000 bps for different lengths of copy number variations. 5000, 10,000 and 25,000 bps were still considered for W (bin sizes). For each setting, we still repeated our simulation and analysis for 5000 times.

**Table 1:** Simulation based performance comparison between the distance-type data and the count-type data (for the detection power; W is the bin size)

*p*-value cutoff	Distance-type data	Count-type data		
		W = 25,000 bps	W = 10,000 bps	W = 5000 bps
Region length L = 50,000 bps				
0.05	28.9%	0	12.8%	16.1%
0.01	12.8%	0	0.1%	7.1%
0.001	3.4%	0	0	2.1%
0.0001	0.8%	0	0	0.3%
0.00001	0.1%	0	0	0
0.000001	0	0	0	0
Region length L = 200,000 bps				
0.05	73.8%	62.4%	68.2%	64.5%
0.01	59.4%	42.8%	50.3%	52.9%
0.001	40.5%	20.2%	30.5%	34.5%
0.0001	26.6%	0	14.3%	24.1%
0.00001	15.3%	0	8.0%	11.3%
0.000001	8.7%	0	1.6%	5.7%
Region length L = 1,000,000 bps				
0.05	97.6%	97.0%	96.8%	96.6%
0.01	94.7%	94.0%	94.1%	94.0%
0.001	89.7%	88.6%	90.1%	90.6%
0.0001	85.3%	83.0%	84.9%	85.9%
0.00001	80.7%	72.1%	80.2%	80.7%
0.000001	75.8%	62.8%	72.7%	75.7%

For each simulation repetition, if the returned *p*-value was lower than the cutoff value, then we calculated the difference between the identified and true change location; otherwise, we considered the difference as no-detection (ND). Then, we would evaluate whether the overall difference based on the distance-type data was lower than the overall difference based on the count-type data. (Notice that a lower difference means a more accurate detection.) Statistically, the traditional Wilcoxon-Mann-Whitney rank sum test would be an appropriate choice (for testing “distance-type based difference” < “count-type based difference”). In the test, for these ND differences, we changed them to be a value larger than any observed differences as no-detection (ND) would be the worst scenario.

### Right censored geometric distribution

Suppose *Y* is a random variable (e.g. distance-type measure) with a right censored geometric distribution with *p* as the probability of success. Notice that a geometric distribution can be considered as a model for the number of failures until first success. Let *T* be a given constant for the censoring value (e.g. the value for artificial right censoring). The indicator function *δ* = 1 if *y* ≥ *T* and *δ* = 0 if *y* < *T*. The probability distribution function of right censored geometric distribution is given by:

1 $$ f(y)={\left(p{\left(1-p\right)}^y\right)}^{\delta }{\left({\left(1-p\right)}^{\left(T+1\right)}\right)}^{\left(1-\delta \right)} $$

*Mixture of Right Censored Geometric Distribution.*

Based on the above right censored geometric distribution, a mixture of right censored geometric distribution can be described. Let *Y*_1_, … , *Y*_*n*_ denote a random sample of size *n*. Each mixture component is a right censored geometric distribution given by Eq. (1) where *p*_*j*_ is the success probability for the *j*-th component. The probability distribution function of a mixture of *g* components is given by:

2 $$ f\left(y,{\pi}_1,{\pi}_2,\dots, {\pi}_g\right)={\sum}_{j=1}^g{\pi}_j{f}_j(y) $$

where

$$ {f}_j(y)={\left({p}_j{\left(1-{p}_j\right)}^y\right)}^{\delta }{\left({\left(1-{p}_j\right)}^{\left(T+1\right)}\right)}^{\left(1-\delta \right)} $$

*π*_1_, *π*_2_, … , *π*_*g*_ are the component proportions subject to the constraint $\sum \limits_{j=1}^g{\pi}_j=1\kern0.5em$and all *π*_*j*_ ∈ [0, 1], all *p*_*j*_ ∈ [0, 1]. The indicator function *δ* = 1 if *y* ≥ *T* and *δ* = 0 if *y* < *T*. All the sample data are (artificially) censored at the same value *T*.

### Mixture of right censured geometric distribution based GLMs

Based on the above mixture of right censored geometric distribution, a mixture of right censored geometric distribution based GLMs can be described. Eq. (2) can be extended to a finite mixture of *g*-component GLMs. Let *y*_1_, … , *y*_*n*_ be *n* independent observations of the response variable. For each *y*_i_, there is a covariate *x*_i_. *π*_1_, … , *π*_*g*_ are the component proportions as defined above. The mixture is given by:

3 $$ f\left(y;x\right)={\sum}_{j=1}^g{\pi}_j{f}_j\left(y;x\right) $$

where *f*_*j*_(*y*; *x*) = (*p*_*j*_(*x*)(1 − *p*_*j*_(*x*))^*y*^)^*δ*^((1 − *p*_*j*_(*x*))^(*T* + 1)^)^(1 − *δ*)^. Notice that each *p*_*j*_ is a function of *x*. We first define *μ*_*j*_ = (1 − *p*_*j*_)/*p*_*j*_ and then we use a link function *η*_*j*_ = log(*μ*_*j*_) = *β*_*j*0_ + *β*_1_*x* for *j* = 1, … , *g*. Here, *β*_1_ is common for all different components since it represents the GC-content effect. Furthermore, the component proportions *π*_1_, … , *π*_*g*_ do not depend on the covariate since they are considered as global parameters (not local). Therefore, the vector *Ψ* of parameters is given by Ψ = (*π*_1_,  … , *π*_*g* − 1_, *β*_10_,  … , *β*_*g*0_, *β*_1_). The intercepts (*β*_10_,  … , *β*_*g*0_) are different for different distribution components.

### Estimation procedure for mixture GLM based on geometric distribution

The log-likelihood based on Eq. (3) is given by

$$ l\left(\uppsi \right)=\log L\left(\uppsi \right)={\sum}_{i=1}^n\log\;\left[{\sum}_{j=1}^g{\pi}_j\;{f}_j\left({y}_i;{x}_i\right)\right]. $$

The EM algorithm [15] can be used to obtain the maximum likelihood estimate of *Ψ*. For each *y*_*i*_, missing component information can be considered. Accordingly, the vector {*z*_*ij*_} is introduced: *z*_*ij*_ = 1 if *y*_*i*_ belongs to the *j*-th component of the mixture model; *z*_*ij*_ = 0 otherwise (*j* = 1,  … , *g*; *i* = 1,  … , *n*). Then, the log-likelihood of “complete data” is given by:

$$ {l}_C\left(\varPsi \right)=\log {L}_C\left(\varPsi \right)={\sum}_{i=1}^n{\sum}_{j=1}^g{z}_{ij}\left\{\log {\pi}_i+\log {f}_j\left({y}_i;{x}_i\right)\right\}. $$

For the E-step, we calculate:

$$ {\tau}_{ij}=\mathbf{E}\;\left({z}_{ij}\left|y\right.\right)={\pi}_j\;{f}_j\left({y}_i;{x}_i\right)/\left[{\sum}_{h=1}^g{\pi}_h\;{f}_h\;\left({y}_i;{x}_i\right).\right] $$

For the M-step, we first calculate:${\pi}_j={\sum}_{i=1}^n{\tau}_{ij}/n.$

The calculation for (*β*_10_,  … , *β*_*g*0_, *β*_1_) requires a numerical optimization procedure, which is used to solve the following equation system.

$$ \sum \limits_{i=1}^n{\tau}_{ij}\frac{\partial }{\partial {\beta}_{j0}}\log {f}_j\left({y}_i;{x}_i\right)=0\ \mathrm{for}\ j=1,\dots, g $$

and

$$ \sum \limits_{i=1}^n\sum \limits_{j=1}^g{\tau}_{ij}\frac{\partial }{\partial {\beta}_1}\log {f}_j\left({y}_i;{x}_i\right)=0 $$

Based on the Newton-Ralphson method, we have the following iterative equation for ***β*** = (*β*_10_,  … , *β*_*g*0_, *β*_1_):

$$ {\beta}_{(r)}={\left[{X}^T WX\right]}^{-1}{X}^T Wz. $$

The details for this iterative equation as well as the EM algorithm are given in the Additional file 1.

### Number of components

The number of components in the mixture model is determined by a likelihood ratio test (LRT) based approach. Consider two integers *g*_0_ < *g*_1_. We test the null hypothesis *H*_0_ : *g* = *g*_0_ against *H*_1_ : *g* = *g*_1_. We first use the observed data to fit a mixture model with *g*_0_ components and a mixture model with *g*_1_ components. A LRT score can be calculated for the observed data. Then, we can simulate data based on the fitted model with *g*_0_ components (parametric bootstrap). For each set of simulated data, we can calculate the related bootstrapped LRT score. After *B* rounds of parametric bootstrap repetitions, the *p*-value of observed LRT score can be approximately calculated based on *B* bootstrapped LRT scores. (If *p*-value< 0.05, the null hypothesis can be rejected.)

## Results

### Distance-type data vs. count-type data

For the detection power based comparison, our evaluation results are summarized in Table 1. As different p-value cutoff values may be used in different scenarios of copy number variation analysis, we considered 0.05, 0.01, 0.001, 0.0001, 0.00001, and 0.000001. Given a cutoff value, a better approach should show a higher proportion (of *p*-values less than the cutoff value). When the regions are as long as 200 kbps, the advantage of distance based approach (distance-type data) can be clearly observed. It is more difficult to detect changes as the regions become shorter. However, the advantage of distance based approach (distance-type data) becomes even clearer when the regions are as short as 50 kbps. When the regions are as long as 1Mbps, two approaches perform overall similarly. From Table 1, it is also clear that the performance of coverage depth based approach (count-type data) depends on the choice of bin size. This has already been discussed in the literature. As our focus in this study is on the choice between distance-type data or count-type data, we prefer not to discuss the choice of bin size for count-type data.

For the detection accuracy based comparison, our evaluation results are summarized in Table 2. The proportion of the lowest *p*-value less than a given cutoff value is presented for each type of data. The proportion based on the distance-type data is always higher. Then, the one-sided p-value for each pair of comparison test is presented in Table 2. (Notice that, the relationship between one-sided and two-sided *p*-values for Wilcoxon-Mann-Whitney rank sum test is actually simple.) When L is relatively short as 150,000 bps, the detection accuracy based on the distance-type data is always better than the detection of accuracy based on the count-type data. When L is increased to 300,000 bps, the detection accuracy based on the distance-type data is still clearly better when the cutoff value is 0.001 or lower. When L is relatively long as 450,000 bps, the detection accuracy based on the count-type data is better, but the detection accuracy based on the distance-type data is still better when the cutoff value is 0.0001 or lower. (Notice that, low cutoff values are necessarily considered in many practical situations.)

**Table 2:** Simulation based performance comparison between the distance-type data and the count-type data (for the detection accuracy evaluation; W is the bin size)

*p*-value cutoff	Distance-type based proportion	Count-type based proportion, and test p-value for distance-type data vs. count-type data		
		W = 25,000 bps	W = 10,000 bps	W = 5000 bps
Region length L = 150,000 bps				
0.01	68.6%	38.2%, < 10^− 6^	55.0%, 6 × 10^− 6^	57.0%, < 10^− 6^
0.001	42.6%	< 0.1%, < 10^− 6^	28.1%, < 10^− 6^	32.9%, < 10^− 6^
0.0001	24.7%	< 0.1%, < 10^− 6^	9.7%, < 10^− 6^	18.5%, < 10^− 6^
0.00001	13.0%	< 0.1%, < 10^− 6^	3.1%, < 10^− 6^	8.9%, < 10^− 6^
0.000001	6.3%	< 0.1%, < 10^− 6^	0.5%, < 10^− 6^	4.5%, 2 × 10^− 5^
0.0000001	3.0%	< 0.1%, < 10^− 6^	< 0.1%, < 10^− 6^	1.3%, < 10^− 6^
Region length L = 300,000 bps				
0.01	86.4%	73.8%, > 0.99	78.1%, 0.929	78.8%, 7 × 10^− 3^
0.001	70.3%	46.6%, < 10^− 6^	58.8%, < 10^− 6^	62.1%, 2 × 10^− 6^
0.0001	53.4%	26.9%, < 10^− 6^	41.4%, < 10^− 6^	47.5%, 3 × 10^− 5^
0.00001	39.9%	< 0.1%, < 10^− 6^	27.3%, < 10^− 6^	33.6%, < 10^− 6^
0.000001	29.1%	< 0.1%, < 10^− 6^	19.1%, < 10^− 6^	24.2%, < 10^− 6^
0.0000001	20.7%	< 0.1%, < 10^− 6^	10.1%, < 10^− 6^	17.6%, 3 × 10^− 4^
Region length L = 450,000 bps				
0.01	92.0%	85.4%, > 0.99	87.7%, > 0.99	87.6%, 0.214
0.001	81.0%	69.3%, > 0.99	75.2%, 0.493	76.2%, 9 × 10^− 3^
0.0001	69.1%	49.2%, < 10^− 6^	60.9%, 1 × 10^− 3^	64.3%, 3 × 10^− 3^
0.00001	57.5%	35.3%, < 10^− 6^	49.1%, 1 × 10^− 5^	52.6%, 6 × 10^− 4^
0.000001	48.0%	14.7%, < 10^− 6^	38.0%, < 10^− 6^	43.4%, 5 × 10^− 4^
0.0000001	38.9%	5.5%, < 10^− 6^	28.6%, < 10^− 6^	36.1%, 0.041

### Estimation and testing performance for mixture of right censored geometric distributions

**Fig. 2:**
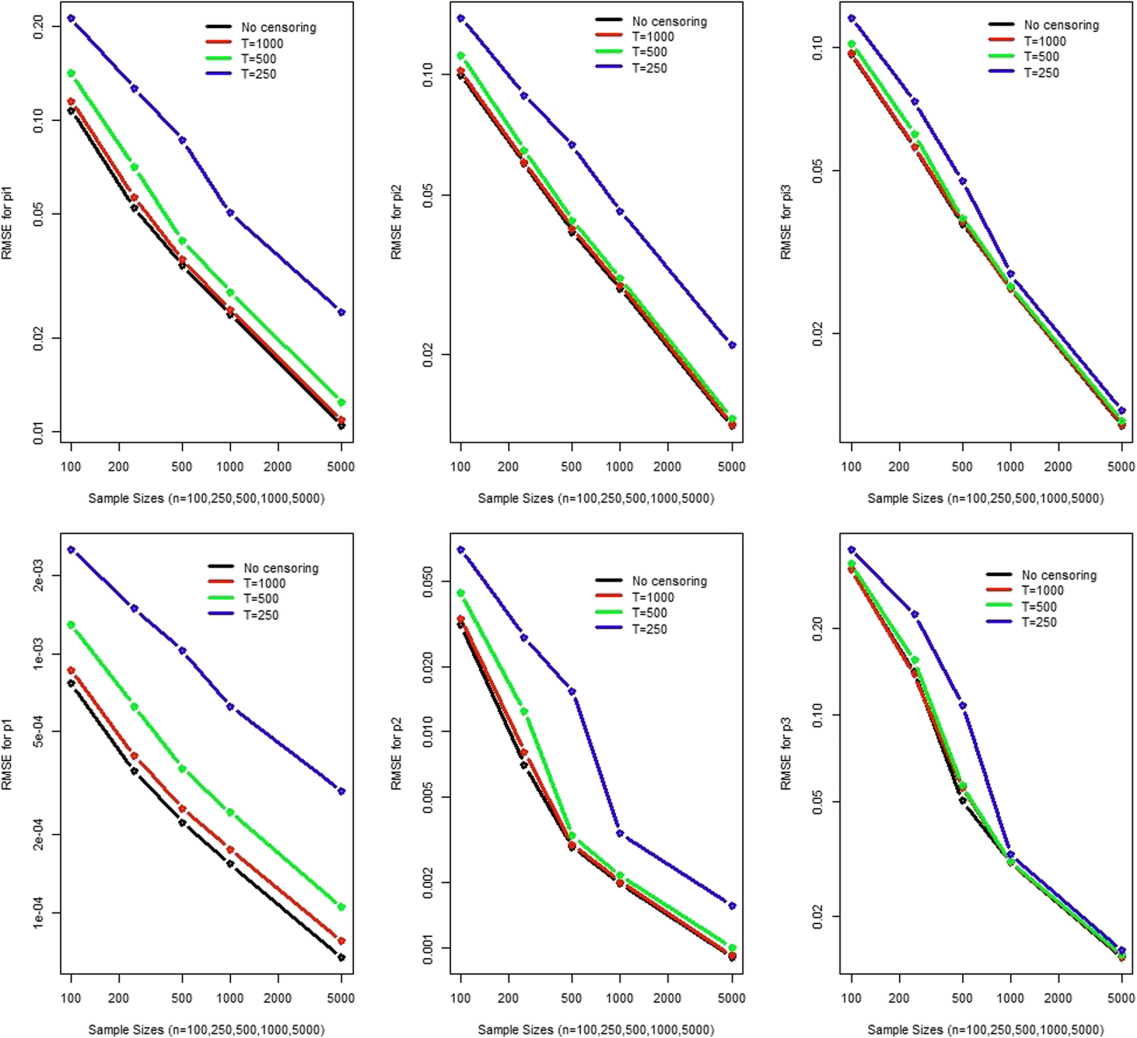
RMSE of the parameter estimates (*π*_1_ = 0.3, *π*_2_ = 0.5, *π*_3_ = 0.2 and *p*_1_ = 0.002, *p*_2_ = 0.02, *p*_3_ = 0.2)

**Fig. 3:**
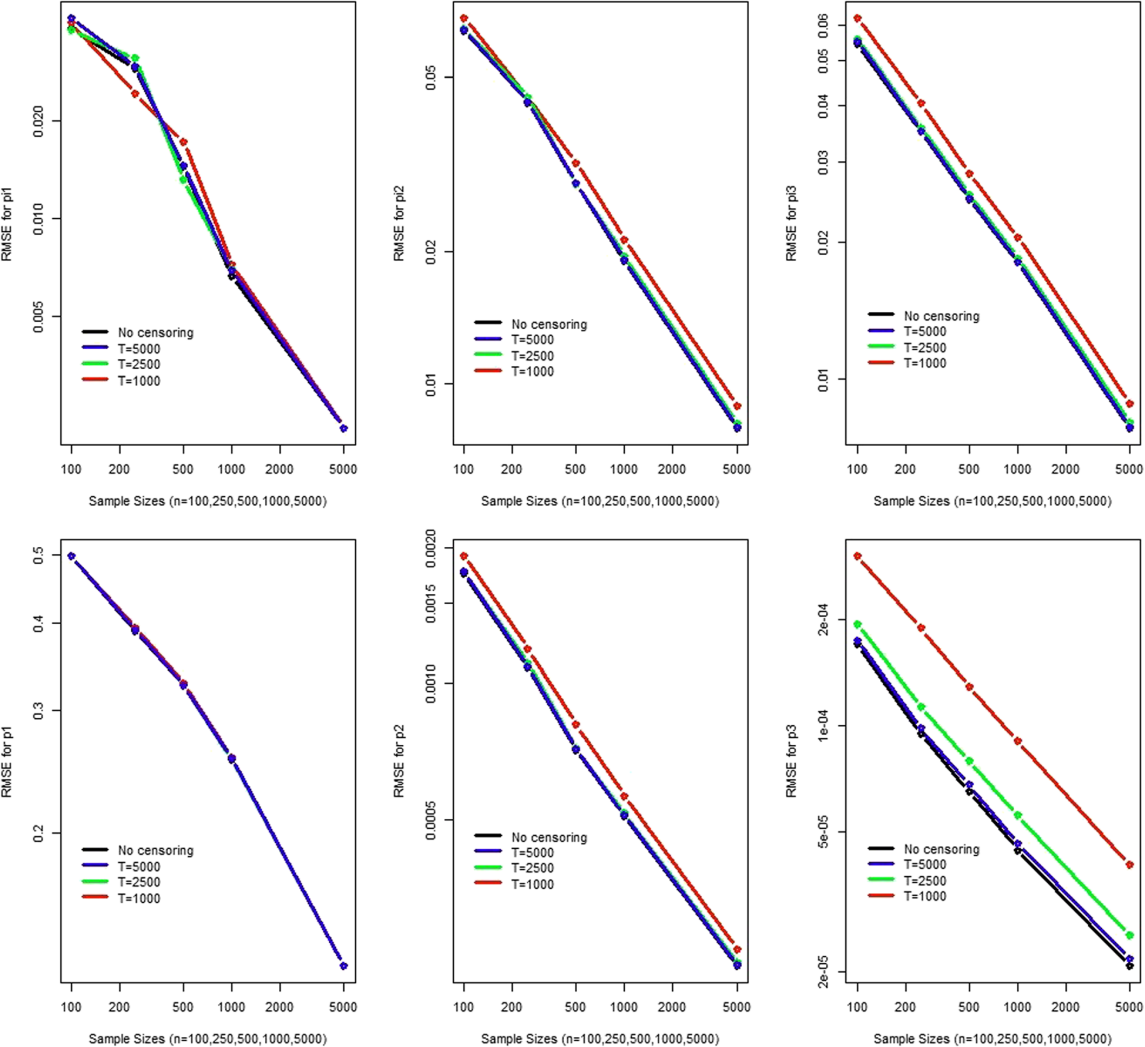
RMSE of the parameter estimates (*π*_1_ = 0.008,  *π*_2_ = 0.754, *π*_3_ = 0.238 and *p*_1_ = 0.999, *p*_2_ = 0.011, *p*_3_ = 0.0006)

We performed a comprehensive simulation study to evaluate the estimation and also the testing performance of our proposed mixture of right censored geometric distribution. To understand estimation when artificial right censoring is applied we simulated a mixture of 3-component geometric with the parameters π_1_ = 0.3, π_2_ = 0.5, π_3_ = 0.2 and p_1_ = 0.002, p_2_ = 0.02, p_3_ = 0.2 (Fig. 2 and Additional file 2: Figures S1-S5). Another simulation was based on a mixture of 3-component geometric with the parameters π_1_ = 0.008,  π_2_ = 0.754, π_3_ = 0.238 and p_1_ = 0.999, p_2_ = 0.011, p_3_ = 0.0006 (Fig. 3 and Additional file 2: Figure S6-S10). For estimation performance evaluation, we simulated 1000 times with 100, 250, 500, 1000 and 5000 samples each with no censoring, censoring at 250, 500 and 1000. We evaluated the bias, variance and root mean square error and used boxplots to summarize the findings (most figures provided as supplementary materials).

The standard deviation and the root mean square error (RMSE, Figs. 2 and 3) decreases with sample size and the censoring value. The RMSE combines both bias and variance. It shows a steady decline as the sample size increases. It also decreases with as the censoring value for observations increases. However for the second simulation, for p_1_ = 0.999 we observed that the median consistently underestimated the true parameter value. Therefore, we need to be cautious when the true parameter value is close to 1. The inter-quartile range becomes narrower with increasing sample size and the artificial censoring value of the observations as well. Overall, both simulation studies support the use of right censored geometric distribution for DNA-seq data.

Furthermore, we have evaluated the effect of censoring on the performance of hypothesis testing. A data set can be simulated as above and we can test the hypothesis of 3 components vs. 4 based on 500 parametric bootstrap p-values. These steps can be repeated 500 times for each case (sample size and censoring value) to generate the empirical distribution of p-values. The empirical distribution plots for these p-values show uniform distribution like patterns (results not shown). Therefore, the hypothesis testing p-values can be appropriately calculated by the parametric bootstrap procedure.

### Estimation and testing performance for mixture GLM where response variable has right censored geometric distributions

To understand estimation and testing performance when artificial right censoring is applied in the case of a mixture GLM, we simulated a mixture of 3-component GLM with the parameters π_1_ = 0.3, π_2_ = 0.5,  π_3_ = 0.2 and β_10_ = 6.0,  β_20_ = 3.6, β_30_ = 1, 1, β_1_ = 2.0. The choice of the component proportions are the same as above and the choice of β_10_, β_20_, β_30_ was based on setting β_1_ = 2.0 and using p_1_ = 0.002, p_2_ = 0.02, p_3_ = 0.2 as above (consistent with a previous simulation). For estimation performance evaluation, we simulated 1000 times with 10,000 samples each with no censoring (NC), censoring at 570, 937, and 1309 respectively (Additional file 2: Figure S11-S12). The censoring values were set such that approximately on average about 10, 5 and 2.5% of the data is censored (consistent with our previous simulations; also about 1–3% in our application). We generated the x-variable from the Uniform [0, 0.25] distribution. A second simulation was based on a mixture of 3-component geometric with the parameters π_1_ = 0.3, π_2_ = 0.5,  π_3_ = 0.2 and β_10_ = 6.2,  β_20_ = 3.9, β_30_ = 1.4,  β_1_ = 0.1. The group proportions were set according to the same strategy as explained above (based on β_1_ = 0.1). For estimation performance evaluation, we simulated 1000 times with 10,000 samples each with no censoring (NC), censoring at 1241, 895 and 549 (Additional file 2: Figure S13-S14). The censoring values were also set according to the same strategy as explained above. We evaluated the bias, variance and root mean square error (results not shown) and used boxplots to summarize the findings (most figures provided as supplementary materials).

The standard deviation and the root mean square error (RMSE) decreases with the increasing censoring value. The RMSE also decreases as the artificial censoring value for observations increases. The median gets closer to the true value with the increasing censoring value of the observations. The inter-quartile range gets narrower with increasing sample size and the censoring value of the observations as well. Overall, both simulation studies support the use of right censoring in mixture GLM.

Furthermore, we have evaluated the effect of censoring on the testing performance. A data set can be simulated as above and we can test the hypothesis of 3 components vs. 4 based on 100 parametric bootstrap p-values. These steps can be repeated 200 times for the first simulation scenario or 100 times for the second simulation scenario (also for each censoring value) to generate the empirical distribution of the *p*-values. (The number of parametric bootstraps was limited to 200 or 100 due to computational burden as this evaluation took much longer time in computing.) Additional file 2: Figure S15 (for the first simulation scenario) and Additional file 2: Figure S16 (for the second simulation scenario) show the empirical distribution plots for these p-values. Uniform distribution like patterns can be observed. Therefore, the hypothesis testing p-values can be appropriately calculated by the parametric bootstrap procedure.

### Application to sequencing data

We considered an application to the DNA-seq data from Chiang et al. [1]. The data set was made publicly available and we selected a normal cell line and the related tumor cell line (HCC1954) for breast carcinoma. Furthermore, we focused on chromosome 9 for an illustration of our method. The value 5000 (bps) was used for the artificial right censoring (we observed at most 1–3% distance data greater than 5000 bps so most of the distance data were relatively short).

A 4-component mixture of right censored geometric distribution based generalized linear models was estimated for the normal sample and then for the tumor sample (Table 3). GC-content adjustment was considered. (We downloaded the 50Kbps-window based GC-content data from http://bioinfo-out.curie.fr/projects/freec .) Notice that, as the GC-content data were based on a reference genome, the model coefficient for GC-content was first estimated based on the normal sample data and then fixed in the model for tumor sample data (all the other model parameters were estimated separately for different sample data). This approach (pseudo-maximum likelihood estimation) was based on the work by Gong and Samaniego [16]. (About the number of mixture components, we could not perform the related hypothesis testing because the related computing could be not afforded. However, we conducted a preliminary data analysis based on the mixture of right censored geometric distributions without considering any covariates, in which we could performed hypothesis testing on the number of mixture components and four distinct components were confirmed. Therefore, we reasonably assumed 4-component for the mixture of GLMs.)

**Table 3:** Parameter estimates from mixture of GLM (censored at 5000 bps)

Parameter	Tumor	Normal
*π*_1_	0.0065	0.0064
*π*_2_	0.1802	0.0359
*π*_3_	0.5414	0.5763
*π*_4_	0.2719	0.3814
*β*_10_	−1.106	−2.021
*β*_20_	6.757	6.691
*β*_30_	8.240	8.681
*β*_40_	9.268	9.457
*β*_1_	−4.428	−4.428

Inverse normal transformations (INTs) have been widely considered in practice. The purpose is to make the (transformed) data more similar to normal distributions. Both rank-based INTs and non-rank based INTs can be considered [17–26]. We considered the following INT. Based on our estimated model (for normal or tumor sample, see Table 3), the cumulative distribution function (CDF) for an observations *y* (not artificially right censored) was calculated by:

4 $$ F\left(y;x\right)={\sum}_{j=1}^g{\pi}_j{F}_j\left(y;x\right) $$

where *F*_*j*_ was the CDF based on *f*_*j*_ given in Eq. (3). Then, an inverse normal distribution function was applied. Through this INT, we obtain the distance-type measure related INT values.

**Fig. 4:**
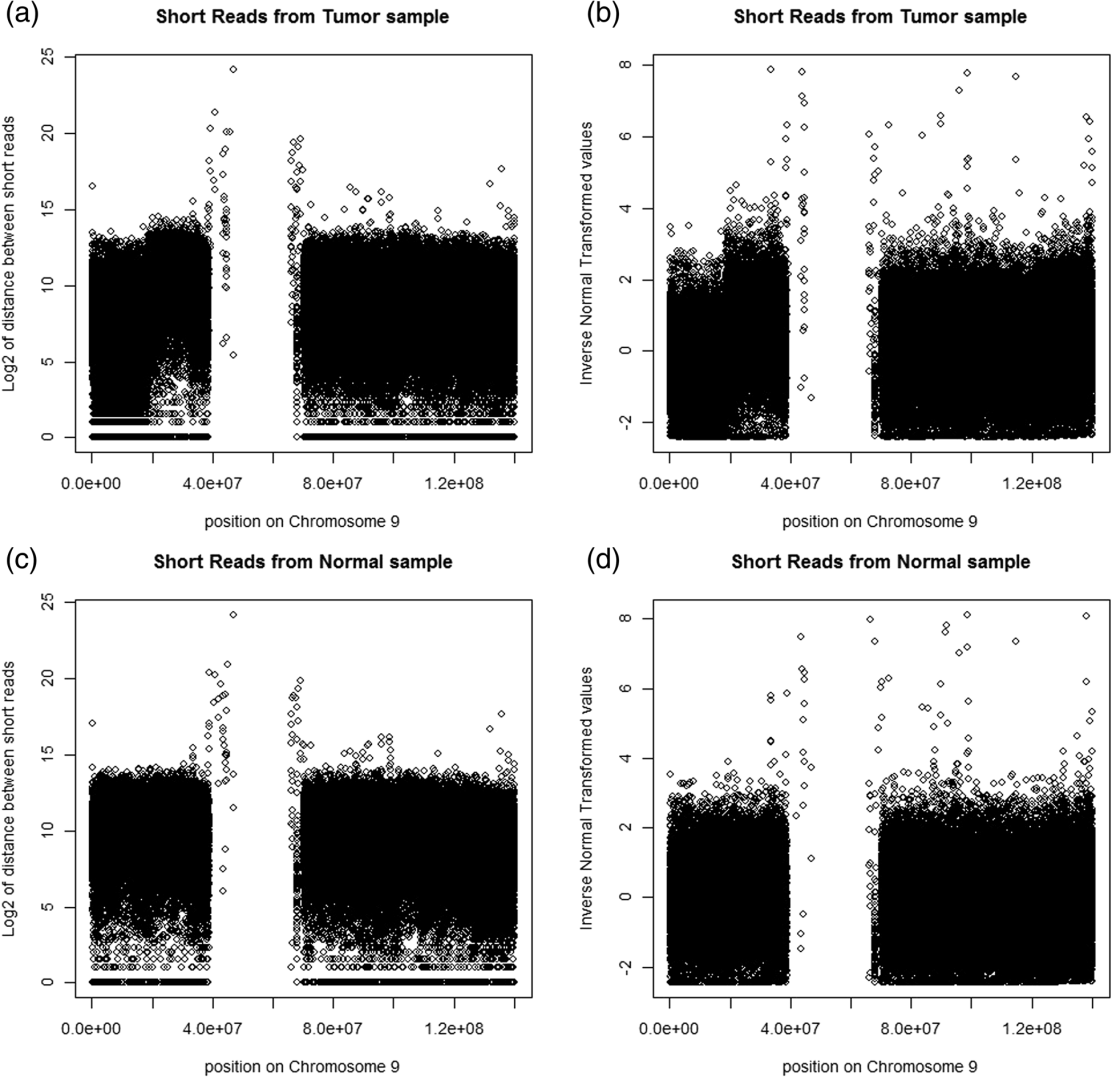
Plots of log_2_ of the distance and INT of the cumulative distribution. (**a**) Plot of log_2_ of the distance between short reads for the tumor sample against the position on chromosome 9; (**b**) Plot of inverse normal transform of the cumulative distribution for the tumor sample versus the position on chromosome 9; (**c**) Plot of log_2_ of the distance between short reads for the normal sample against the position on chromosome 9; (**d**) Plot of inverse normal transform of the cumulative-distribution for the normal sample versus the position on chromosome 9

The comparison between the distance data (log2 of distance+ 1) and the related INT values (inverse normal transformed values; not related to artificially right censored distance data) is shown in Fig. 4. Some uneven patterns can be visualized from the plots of distance data. These were not observed from the plots of z-values. Therefore, the GC-content adjustments have been well considered in our method.

**Fig. 5:**
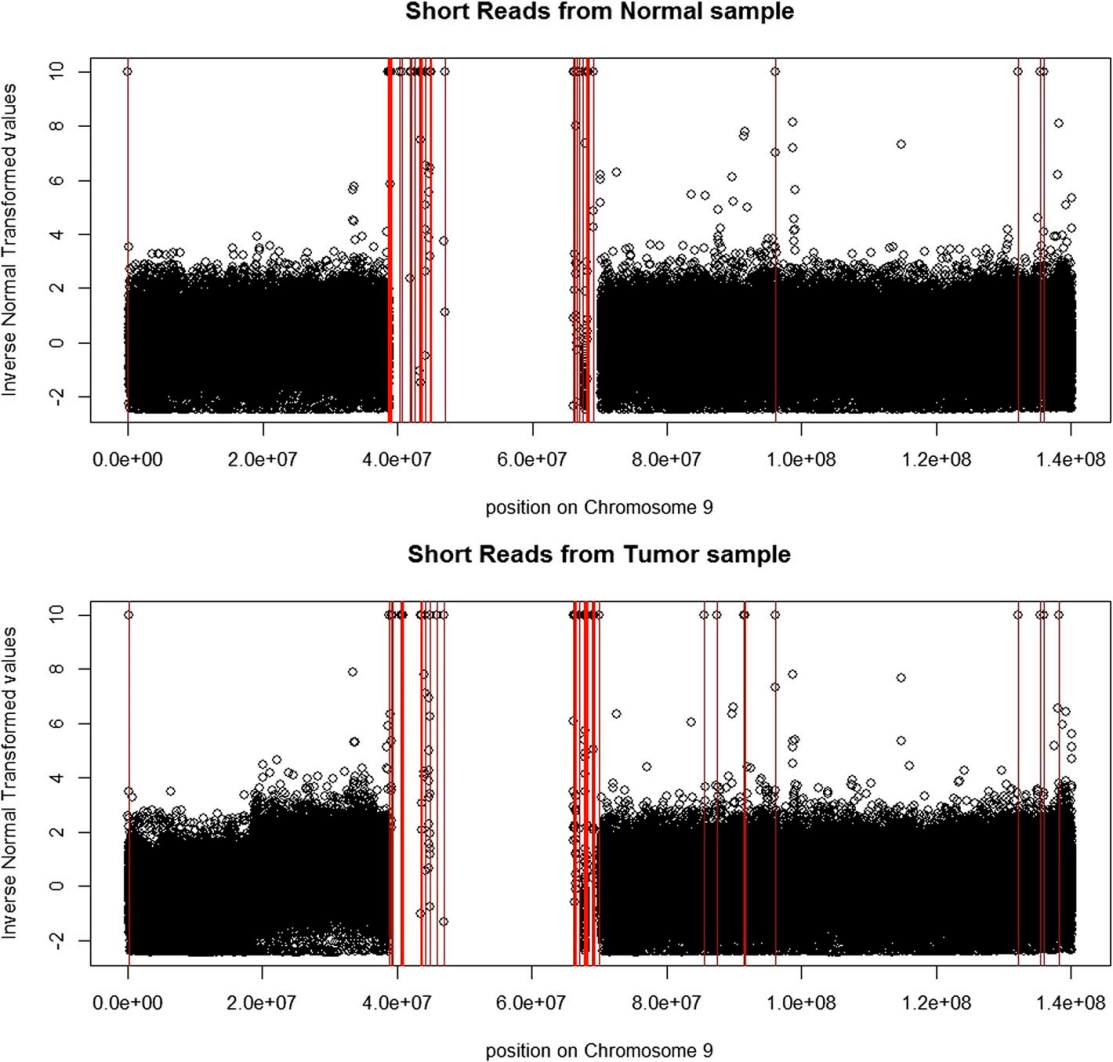
Plots of INT of the cumulative distribution against the position on chromosome 9, represented as bps

Then, for the INT values related to these artificially right censored distance data, we simply assign a value 10 (larger than any INT values in Fig. 4). In Fig. 5, more details can be visualized for INT values. Notice that censored observations are highlighted by red vertical lines (there are 31 instances for the normal sample and 35 instances for the tumor sample). Some differences on the region around 200 K-38 M bps (chromosome 9) can be observed and we performed a change-point analysis for this region. (The normality was checked for INT values. We randomly selected regions with length 100 K bps for both the normal and tumor samples and generated the related quantile-quantile plots [Q-Q plots, Additional file 2: Figure S17-S18]. Then, we assumed that INT values were normally distributed, which was helpful to avoid the permutation based *p*-value calculation [27] in the next change-point analysis.)

For a change-point analysis of the above selected region (as an illustration), we considered a recursive combination approach [28]. For each round of recursion, at a given level α_C_, any two adjacent blocks of INT values with two-sample test *p*-values larger than α_C_ were selected. Among them, the combination to generate the largest overall likelihood was applied. The recursion stopped when all the test *p*-values were less than α_C_. Considering the large number of two-sample comparisons, we used 50 K bps bins to group INT values before the change-point analysis. We set α_C_ = 0.05/(m ∗ (m − 1)/2), where m is the number of bins (767 for normal sample and 772 for tumor sample).

**Fig. 6:**
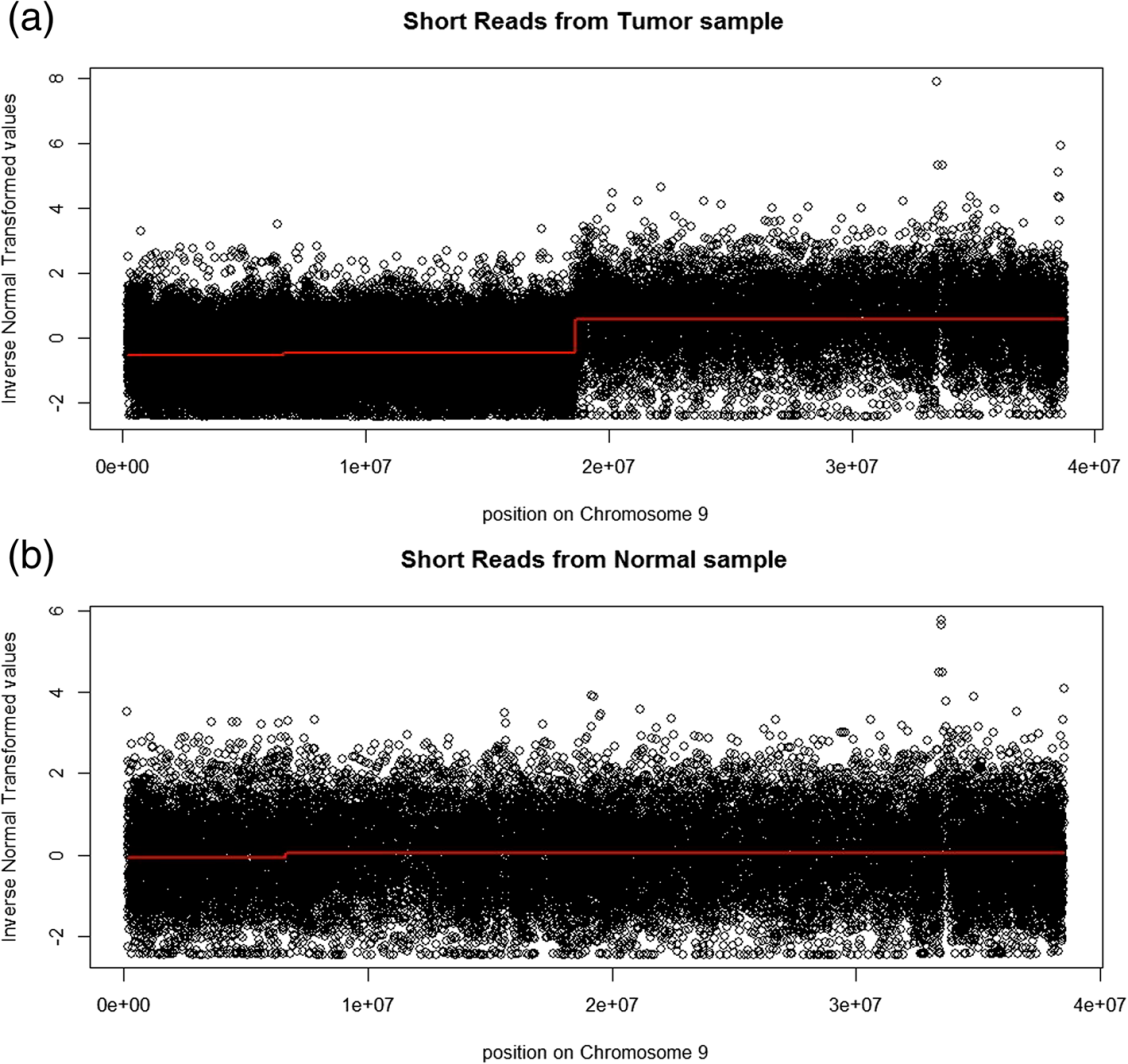
INT of the cumulative distribution with the estimated mean represented by the red line. (**a**) Plot for the tumor sample where the red line is the estimated mean from the recursive combination algorithm; (**b**) plot for the normal sample where the red line is the estimated mean from the recursive combination algorithm

The change-point analysis results are shown in Fig. 6. For both normal and tumor samples, there is a change-point around 6-7 M bps. However, the magnitude of changes is small. This detection is unlikely to be biologically relevant. For the tumor sample, there is an additional change-point around 18-19 M bps, which implies a clear deletion (larger distance related to less DNA copy number). Cytobands have been widely considered in biomedical studies [29]. A search in UCSC data base (http://www.genome.ucsc.edu/cgi-bin/hgTracks) shows that the cytobands p13.2, p13.3, p21.1, p21.2, p21.3 and p22.1 are related to this detected region. To understand biological significance of this detected deletion, a literature search shows that this region has been investigated by many studies on the relationship between these cytobands and breast cancer [30, 31].

## Discussion

Count-type data analysis is a widely used approach for DNA-seq data. It requires either a fixed or a sliding window procedure for generating count-type data, and useful information can be reduced after this data pre-processing. In this study, we proposed to analyze DNA-seq data based on the related distance-type measure. Our experimental data based simulation study confirmed the advantages of distance-type measure approach in both detection power and detection accuracy. Furthermore, we proposed a mixture of generalized linear models for analyzing the recent DNA-seq data. Our model was based on the right censored geometric distribution. According to the mathematical details provided in the Additional file 1, our method is linear in term of the number of sequencing reads. Therefore, our method scales well. The validity and usefulness of this approach were demonstrated by the estimation and testing performances in our simulation study as well as an application to experimental data. The selection of the censoring value was based on that 1–3% of observed distance data were larger than 5000 bps. The optimization of the censoring value is an interesting research topic and we will investigate this in our future study. Our distance-based approach is novel with a clear advantage: it does not require either a fixed or a sliding window procedure for generating count-type data.

Similar as the exponential distribution (for continuous measurements), which is widely used to model waiting time processes, the geometric distribution (for discrete measurements) is also a memoryless probability distribution. The artificial right-censoring has been proposed to overcome the difficult in modeling extreme distance values. (For example, it is difficult to obtain sequencing reads near centromere regions.) Due to different biological properties in different genomic regions, which results in different distance distributions, different geometric components are necessary to model the data. As one geometric distribution does not well fit the distance data, a mixture of right-censored geometric distributions has been proposed. To understand the connection between a probability distribution component and its related underling biological properties, we need further statistical and biological investigations, which will be an interesting topic for our future research.

In our application, interesting change-points were observed from the tumor cell line data. The impact from GC-content was considered in our analysis. In this study, we chose a set of relatively simple DNA-seq data for the purpose of illustration of our approach. Our approach can also be applied to paired-end DNA-seq data. In practice, either paired-end reads or single-end reads can be considered in sequencing experiments. If only the starting position of each paired-end read is considered, then the analysis scenario is equivalent to the analysis of single-end reads. However, this strategy may not well utilize the advantage of paired-end reads. (For example, longer regions can be covered by a paired-end read.) To consider the distance based approach and to utilize the advantage of paired-end reads, we will need to investigate which modifications or extensions are necessary to our method proposed in this study. This will be an interesting topic for our future research.

## Conclusion

With the next generation sequencing technology, a large amount of short DNA sequence reads can be obtained at a genome-wide level. DNA-seq data have been increasingly used in different areas of biomedical studies. Although the count-type based approaches have been widely used for DNA-seq data analysis, a moving window based data pre-processing (or similar) is required with a pre-specified window size. Useful information can be reduced after the data pre-processing. In this study, we have developed a distance-type measure based method. Distances are measured in base pairs (bps) between two adjacent alignments of short reads mapped to a reference genome. This is a novel approach. Its advantages have been demonstrated by our simulation studies and its practical usefulness has been illustrated by an experimental data application.

## Additional files

Additional file 1: Mathematical details. (PDF 271 kb)

Additional file 2: Supplemental figures. (PDF 1697 kb)

## Abbreviations

CNA: copy number alteration
CNV: copy number variation
DNA-seq: short DNA sequence
E-M algorithm: Expectation-Maximization algorithm
GLM: generalized linear model
INT: Inverse normal transformation
LRT: likelihood ratio test
Q-Q plot: quantile-quantile plot
RMSE: root mean square error

## References

[1] Chiang DY (2009). High-resolution mapping of copy-number alterations with massively parallel sequencing. Nat Methods.

[2] Miller CA (2011). ReadDepth: a parallel R package for detecting copy number alterations from short sequencing reads. PLoS One.

[3] Xie R (2011). Detecting structural variations in the human genome using next-generation sequencing. Brief Funct Genomics.

[4] Kim TM (2010). rSW-seq: algorithm for detection of copy number alterations in deep sequencing data. BMC Bioinformatics.

[5] Krishnan NM (2012). COPS: a sensitive and accurate tool for detecting somatic copy number alterations using short-read sequence data from paired samples. PLoS One.

[6] Korbel JO (2007). Paired-end mapping reveals extensive structural variation in the human genome. Science.

[7] Sudmant PH (2010). Diversity of human copy number variation and multicopy genes. Science.

[8] Wang TL (2002). Digital karyotyping. Proc Natl Acad Sci U S A.

[9] Leary RJ (2007). Digital karyotyping. Nat protocols.

[10] Morozova O (2008). Applications of next-generation sequencing technologies in functional genomics. Genomics.

[11] Shen JJ, Zhang N (2012). Change-point model on nonhomogeneous Poisson process with application in copy number profiling by next-generation DNA sequencing. Ann Appl Stat.

[12] Benjamini Y, Speed TP (2012). Summarizing and correcting the GC content bias in high-throughput sequencing. Nucleic Acids Res.

[13] Aitkin M, Rubin DB (1985). Estimation and hypothesis testing in finite mixture models. Royal Statistical Society.

[14] McLachlan G, Peel D (2002). Finite Mixture Models.

[15] Dempster AP, Laird NM, Rubin DB (1977). Maximum likelihood from incomplete data via the EM algorithm. J R Stat Soc Ser B.

[16] Gong G, Samaniego FJ (1981). Pseudo maximum likelihood estimation: theory and applications. Ann Stat.

[17] Ashton GC, Borecki IB (1987). Further evidence for a gene influencing spatial ability. Behav Genet.

[18] Silverman EK (1990). Biochemical intermediates in alpha 1-antitrypsin deficiency: residual family resemblance for total alpha 1-antitrypsin, oxidized alpha 1-antitrypsin, and immunoglobulin E after adjustment for the effect of the pi locus. Genet Epidemiol.

[19] Tzou GG (1991). Classification of beef calves as protein-deficient or thermally stressed by discriminant analysis of blood constituents. J Anim Sci.

[20] Martin LJ, Crawford MH (1998). Genetic and environmental components of thyroxine variation in Mennonites from Kansas and Nebraska. Hum Biol.

[21] Basrak B (2004). Copulas in QTL mapping. Behav Genet.

[22] Przybyla-Zawislak BD (2005). Identification of rat hippocampal mRNAs altered by the mitochondrial toxicant, 3-NPA. Ann N Y Acad Sci.

[23] Li M (2006). Quantitative trait linkage analysis using Gaussian copulas. Genetics.

[24] Hicks BM (2007). Genes mediate the association between P3 amplitude and externalizing disorders. Psychophysiology.

[25] Kraja AT (2007). Rheumatoid arthritis, item response theory, Blom transformation, and mixed models. BMC Proc.

[26] Knoll J, Ejeta G (2008). Marker-assisted selection for early-season cold tolerance in sorghum: QTL validation across populations and environments. Theor Appl Genet.

[27] Lai Y (2012). Change-point analysis of paired allele-specific copy number variation data. J Comput Biol.

[28] Lai Y (2011). On the adaptive partition approach to the detection of multiple change-points. PLoS One.

[29] Furey TS, Haussler D (2003). Integration of the cytogenetic map with the draft human genome sequence. Hum Mol Genet.

[30] Brenner AJ, Aldaz CM (1995). Chromosome 9p allelic loss and p16/CDKN2 in breast cancer and evidence of p16 inactivation in immortal breast epithelial cells. Cancer Res.

[31] Nessling M (2005). Candidate genes in breast cancer revealed by microarray-based comparative genomic hybridization of archived tissue. Cancer Res.

