# Supplementary material for: A distance-type measure approach to the analysis of copy number variation in DNA sequencing data
Source: BMC Genomics. 2019 Apr 4;20(Suppl 2):195. doi: 10.1186/s12864-019-5491-x (PMC6456939; doi:10.1186/s12864-019-5491-x)
Supplement: Supplementary file 1 — Mathematical details. (PDF 271 kb) [file 12864_2019_5491_MOESM1_ESM.pdf]

Mixture of Right Censored Geometric Distribution

The probability density function of a mixture of k-component right censored univariate geometric distributions is given by:

$$f(y,\pi_1,\pi_2,\dots,\pi_g)=\sum_{j=1}^{g}\pi_j f_j(y)$$

where (5)

$$f_j(y)=\left(p_j(1-p_j)^y\right)^{\delta}\left(\left(1-p_j\right)^{(T+1)}\right)^{(1-\delta)}.$$

The $\pi_1,\pi_2,\dots,\pi_g$ are the proportion of each component and $\delta=1\ if\ y\le T$ and $0\ if\ y>T$ where the samples are censored at a fixed constant T. We consider Type I right censored distributions here. The objective is to determine the unknown parameters $p_j, j=1,2,\dots,g$ and the components $\pi_1,\pi_2,\dots,\pi_g$ subject to the constraint $\sum_{j=1}^{g}\pi_j=1$, all $\pi_j\in[0,1]$, and all $p_j\in[0,1]$. The likelihood and the log likelihood functions based on n observations $y_1,y_2,\dots,y_n$ from the mixture distributions in Equation (5) are given by:

$$L(\pi_1,\pi_2,\dots,\pi_g,p_1,p_2,\dots,p_g)=\prod_{i=1}^{n}\left[\sum_{j=1}^{g}\pi_j f_j(y_i)\right]$$

$$l(\pi_1,\pi_2,\dots,\pi_g,p_1,p_2,\dots,p_g)=\sum_{i=1}^{n}log\left[\sum_{j=1}^{g}\pi_j f_j(y_i)\right]. \quad (6)$$

We maximize Equation (6) subject to the constraint $\sum_{j=1}^{g}\pi_j=1$. Introducing a Lagrange multiplier λ for the constraint, we have to find stationary points of the function

$$l^*(\pi_1,\pi_2,\dots,\pi_g,p_1,p_2,\dots,p_g)=\ l(\pi_1,\pi_2,\dots,\pi_g,p_1,p_2,\dots,p_g)-\lambda\left[\sum_{j=1}^{g}\pi_j-1\right].$$

The partial derivative of $l^*$with respect to π_h is

$$\frac{\partial l^*}{\partial\pi_h}=\sum_{i=1}^{n}\frac{f_h(y_i)}{\sum_{j=1}^{g}\pi_j f_j(y_i)}-\lambda,\qquad h=1,2,\dots,g \quad (7)$$

Setting Equation (7) equal to zero and multiplying it by π_h(assuming $\pi_h\neq 0$) yields

$$\lambda\hat{\pi}_h=\sum_{i=1}^{n}\frac{\hat{\pi}_h f_h(y_i)}{\sum_{j=1}^{g}\hat{\pi}_j f_j(y_i)}\qquad h=1,2,\dots,g. \quad (8)$$

Introducing the symbol corresponding to the posterior probability of the ith observation coming from component h:

$$\hat{z}_{hi}=\sum_{i=1}^{n}\frac{\hat{\pi}_h f_h(y_i)}{\sum_{j=1}^{g}\hat{\pi}_j f_j(y_i)}\qquad h=1,2,\dots,g;\quad i=1,2,\dots,n.$$

Thus Equation (8) reads as

$$\lambda\hat{\pi}_h=\sum_{i=1}^{n}\hat{z}_{hi}\qquad h=1,2,\dots,g.$$

Adding Equation (8) for $h=1,2,\dots,k$, and using the constraint $\sum_{j=1}^{g}\pi_j=1$, we obtain $\lambda=n$. Substituting this in Equation (8) and from Equation (1) we get

$$\hat{\pi}_h=\frac{1}{n}\sum_{i=1}^{n}\hat{z}_{hi}\qquad h=1,2,\dots,g$$

$$=\frac{1}{n}\sum_{i=1}^{n}\frac{\hat{\pi}_h(\hat{p}_h(1-\hat{p}_h)^{y_i})^{\delta_i}\left((1-\hat{p}_h)^{(T+1)}\right)^{(1-\delta_i)}}{\sum_{j=1}^{g}\hat{\pi}_j\left(\hat{p}_j(1-\hat{p}_j)^{y_i}\right)^{\delta_i}\left((1-\hat{p}_j)^{(T+1)}\right)^{(1-\delta_i)}}\qquad h=1,2,\dots,g. \quad (9)$$

Equation (9) states that the prior probability for the hth component is to be estimated as the average of the posterior probabilities of all n observations for this component.

Now turning to right censored geometric mixtures and considering only the univariate case where the density of the mixture is denoted by Equation (5). Proceeding with the partial derivatives of log likelihood function in (6) with respect to the unknown parameters p_h for $h=1,2,\dots,k$ we get

$$\frac{\partial l}{\partial p_h} = \sum_{i=1}^{n} \frac{\partial log f(y_i)}{\partial p_h}$$
$$= \sum_{i=1}^{n} \frac{1}{f(y_i)} \frac{\partial f(y_i)}{\partial p_h}$$
$$= \sum_{i=1}^{n} \frac{1}{f(y_i)} \pi_h \frac{\partial f_h(y_i)}{\partial p_h}.$$

Because p_h appears only in the hth component density. Using the fact that for a nonnegative function $g(u)$,

$$\frac{\partial g(u)}{\partial u} = g(u) \frac{\partial log g(u)}{\partial u},$$

we obtain

$$\frac{\partial l}{\partial p_h} = \sum_{i=1}^{n} \frac{\pi_h f_h(y_i)}{f(y_i)} \frac{\partial log f_h(y_i)}{\partial p_h}. \quad (10)$$

Now the hth density in particular is given by

$$f_h(y_i) = (p_h(1-p_h)^{y_i})^{\delta_i} \left((1-p_h)^{(T+1)}\right)^{(1-\delta_i)}.$$

And the log of the density is

$$log f_h(y_i) = \delta_i log p_h + \delta_i y_i log(1-p_h) + (T+1)(1-\delta_i)\log(1-p_h).$$

Taking the partial derivative of Equation (6) with respect to p_h we get

$$\frac{\partial log f_h(y_i)}{\partial p_h} = \frac{\delta_i}{p_h} - \frac{\delta_i y_i}{(1-p_h)} - \frac{(T+1)(1-\delta_i)}{(1-p_h)} \qquad h = 1,2,\ldots,g. \quad (11)$$

Substituting Equation (11) in Equation (10) and equating it to zero yields

$$\sum_{i=1}^{n} \frac{\pi_h f_h(y_i)}{f(y_i)} \left(\frac{\delta_i}{p_h} - \frac{\delta_i y_i}{(1-p_h)} - \frac{(T+1)(1-\delta_i)}{(1-p_h)}\right) = 0 \qquad h = 1,2,\ldots,g.$$

Using Equation (8) we get

$$\sum_{i=1}^{n} z_{hi} \left(\frac{\delta_i}{p_h} - \frac{\delta_i y_i}{(1-p_h)} - \frac{(T+1)(1-\delta_i)}{(1-p_h)}\right) = 0 \qquad h = 1,2,\ldots,g.$$

Solving the above equation for p_h yields the maximum likelihood estimates of p_h given by

$$\hat{p}_h = \frac{\sum_{i=1}^{n} \hat{z}_{hi}\delta_i}{\sum_{i=1}^{n} \hat{z}_{hi}[(\delta_i + \delta_i y_i) + (T+1)(1-\delta_i)]}, \qquad h = 1,2,\ldots,g.$$

The EM algorithm for maximizing likelihood function with missing data consists of iterated applications of the following two steps:

E-step (Expectation): Replace the complete data log-likelihood by its expectation taken with respect to the missing data given the observed data and using the current parameter values in the calculations of the expectation.

M-Step (Maximization): Solve the likelihood equations for complete data likelihood, using the data substituted in the E-step.

Thus using the methods for EM algorithm discussed above, we can provide an EM algorithm for right censored geometric mixtures as:

Step 0 (initialization): Assigning initial values to the parameters

$\pi_1, \pi_2, \ldots, \pi_g$ and $p_1, p_2, \ldots, p_g$.

Step 1(E-step): set

$$z_{hi} = \frac{\pi_h f_h(y_i)}{f(y_i)} \qquad h = 1,2,\ldots g; i = 1,2,\ldots,n$$

where $f_h(y_i) = (p_h(1-p_h)^{y_i})^{\delta_i}\left((1-p_h)^{(T+1)}\right)^{(1-\delta_i)}$ and

$f(y_i) = \sum_{j=1}^{g} \pi_j f_j(y_i)$.

Step 2 (M-step): set

$$\hat{\pi}_h = \frac{1}{n}\sum_{i=1}^{n} \hat{z}_{hi} \qquad h = 1,2,\dots,g$$

$$= \frac{1}{n}\sum_{i=1}^{n} \frac{\hat{\pi}_h(\hat{p}_h(1-\hat{p}_h)^{y_i})^{\delta_i}\left((1-\hat{p}_h)^{(T+1)}\right)^{(1-\delta_i)}}{\sum_{j=1}^{k} \hat{\pi}_j\left(\hat{p}_j(1-\hat{p}_j)^{y_i}\right)^{\delta_i}\left((1-\hat{p}_j)^{(T+1)}\right)^{(1-\delta_i)}} \qquad h = 1,2,\dots,g$$

and

$$\hat{p}_h = \frac{\sum_{i=1}^{n} \hat{z}_{hi}\delta_i}{\sum_{i=1}^{n} \hat{z}_{hi}[(\delta_i+\delta_i y_i)+(T+1)(1-\delta_i)]}\ , \qquad h = 1,2,\dots,g.$$

Then return to step 1. The iteration continues till convergence.

Mixture of GLM based on right censored geometric distribution

The log-likelihood for Ψ for data under the mixture model is given by

$$\mathrm{LogL}(\Psi) = \sum_{i=1}^{n} \log\left[\sum_{j=1}^{g} \pi_i\, f_j(y_i; p_{ij})\right]$$

Where the individual component densities are given by

$$f_j(y_i; p_{ij}) = \exp\{\delta_j y_j \log(1-p_{ij}) + \delta_j \log(p_{ij}) + (1-\delta_j)(T+1)\log(1-p_{ij})\}$$

And $\mu_{ij} = \frac{1-p_{ij}}{p_{ij}}$ gives $p_{ij} = \frac{1}{1+\mu_{ij}}$. Using the log link function $\eta_{ij} = \log(\mu_{ij}) = X_i\beta$ where X_i is the matrix of covariates for the ith component.

Thus in matrix form

$$X_i = \begin{pmatrix} 0 & 1 & 0 x_{11} \\ \vdots \dots \vdots \dots \vdots & \vdots \\ 0 & 1 & 0 x_{n1} \end{pmatrix}_{n\times(g+1)} ; \beta = \begin{pmatrix} \beta_{10} \\ \beta_{20} \\ \vdots \\ \beta_{g0} \\ \beta_1 \end{pmatrix}_{(g+1)\times 1}$$

The link function can be expressed as $\eta_{ij} = \beta_{i0} + \beta_1 x_{j1}$ and since $\eta_{ij} = \log(\mu_{ij})$ we get $\mu_{ij} = \exp(\eta_{ij}) = \exp(\beta_{i0} + \beta_1 x_{j1})$.

The EM algorithm discussed by Dempster et al., (1977) can be applied to obtain the maximum likelihood estimate of Ψ as in the case of finite mixture of arbitrary distributions. In the incomplete data framework for the application of the EM algorithm to the problem of mixture GLM, each response y_j is viewed as having arisen from one of the g components of the postulated mixture model of GLM. Accordingly for each y_j the vector z_j is introduced as missing data, where $z_{ij} = 1$ or 0 accordingly as y_j does or does not belong to the ith component of the mixture model$(i = 1,\dots,g; j = 1,\dots,n)$. The unobservable indicator vector z_j is taken to be the re-alignment of a random sample of size one from a multinomial distribution, consisting of a single draw on g categories with probabilities $\pi_1,\dots,\pi_g$; $z_1,\dots,z_n$ are independently distributed.

The complete-data log-likelihood is given by

$\mathrm{LogL}_C(\Psi) = \sum_{i=1}^{g} z_{ij}\{\log\pi_i + \log f_i(y_j; p_{ij})\}$.

The EM algorithm applies to the mixture models by treating the z_{ij} as missing data. The unobservable or the missing data in the EM algorithm framework is handled in the E or the Expectation step which takes the conditional expectation of the complete-data log likelihood,

$\log L_c(\Psi)$, given the observed data y, using the current fit for Ψ. Let $\Psi^{(0)}$ be the initial value for Ψ. Thus, the E-step involves the computation of the conditional expectation of $\log L_c(\Psi)$ given y, using $\Psi^{(0)}$written as

$$Q(\Psi;\Psi^{(0)}) = E_{\Psi^{(0)}}\{\log L_c(\Psi)|y\}.$$

It follows from here that on the $(k+1)$th iteration, the E-step involves the calculation of $Q(\Psi;\Psi^{(k)})$, where $\Psi^{(k)}$ is the value of Ψ after the kth EM iteration. Thus,

$$\hat{Z}_{ij} = E_{\Psi^{(k)}}(Z_{ij}|y) = pr_{\Psi^{(k)}}\{Z_{ij} = 1|y\}$$
$$= \tau_i(y_j;\Psi^{(k)}).$$

The quantity $\tau_i(y_j;\Psi^{(k)})$ is the posterior probability that the jth member of the sample with observed value y_j belongs to the ith component of the mixture. Thus on the $(k+1)$th iteration of the EM algorithm, the E-step is easily affected to give the Q-function

$$Q(\Psi,\Psi^{(k)}) = \sum_{i=1}^{g}\sum_{j=1}^{n}\tau_i(y_j;\Psi^{(k)})\{\log\pi_i + \log f_i(y_j;p_{ij})\}$$

where

$$\tau_i(y_j;\Psi^{(k)}) = \frac{\pi_i f_i(y_j;p_{ij}{}^{(k)})}{\sum_{h=1}^{g}\pi_h f_h(y_j;p_{hj}{}^{(k)})}$$

is the current estimate of the posterior probability that the jth response belongs to the ith component given y_j with covariates X_j $(j = 1,\dots,n)$.

M-Step:

Since the mixing proportions $\pi_1,\dots,\pi_g$ do not depend upon any covariates, the updated estimate of $\pi_i$is given by

$$\pi_i^{(k+1)} = \tau_i(y_j;\Psi^{(k)})/n$$

where $\tau_i(y_j;\Psi^{(k)})$ is given as above and the computation of $\beta^{(k+1)}$follows from

$$\sum_{i=1}^{g}\sum_{j=1}^{n}\tau_i(y_j;\Psi^{(k)})\frac{\partial}{\partial\beta}\log f_i(y_j;p_{ij}) = 0$$

which for our case with only one covariate and intercept reduces to

$$\sum_{j=1}^{n}\tau_i(y_j;\Psi^{(k)})\frac{\partial}{\partial\beta_{i0}}\log f_i(y_j;p_{ij}) = 0 \qquad i = 1,\dots,g$$

and

$$\sum_{i=1}^{g}\sum_{j=1}^{n}\tau_i(y_j;\Psi^{(k)})\frac{\partial}{\partial\beta_1}\log f_i(y_j;p_{ij}) = 0.$$

Thus for the right-censored geometric distributions

$$f_i(y_j;p_{ij}) = \exp\{\delta_j y_j\log(1-p_{ij}) + \delta_j\log(p_{ij}) + (1-\delta_j)(T+1)\log(1-p_{ij})\}$$

with link function $\eta_{ij} = \log(\mu_{ij}) = X_i\beta$ and $\mu_{ij} = \frac{1-p_{ij}}{p_{ij}}$ we have

$$f_i(y_j;p_{ij}) = \exp\left\{\delta_j y_j\log\left(\frac{\mu_{ij}}{1+\mu_{ij}}\right) - \delta_j\log(1+\mu_{ij}) + (1-\delta_j)(T+1)\log\left(\frac{\mu_{ij}}{1+\mu_{ij}}\right)\right\}.$$

Which when expressed in terms of β reduces to

$$f_i(y_j;p_{ij}) = \exp\left\{\delta_j y_j(\beta_{i0}+\beta_1 x_{j1}) - \delta_j\log\left(1+\exp(\beta_{i0}+\beta_1 x_{j1})\right) + (1-\delta_j)(T+1)\left[\beta_{i0}+\beta_1 x_{j1} - \log\left(1+\exp(\beta_{i0}+\beta_1 x_{j1})\right)\right]\right\}.$$

Thus the first derivative of the log of the above density is given by

$$\frac{\partial f_i(y_j;p_{ij})}{\partial\beta_{i0}} = \frac{\delta_j(y_j-\mu_j)+(1-\delta_j)(T+1)}{1+\mu_j} \qquad i = 1,\dots,g$$

and

$$\frac{\partial f_i(y_j;p_{ij})}{\partial \beta_1}=\frac{\delta_j(y_j-\mu_{ij})+(1-\delta_j)(T+1)}{1+\mu_{ij}}x_{j1} \quad .$$

Substituting the above derivatives results in the following system of equations

$$\sum_{j=1}^{n}\tau_i(y_j;\Psi^{(k)})\left(\frac{\delta_j(y_j-\mu_{ij})+(1-\delta_j)(T+1)}{1+\mu_{ij}}\right)=0 \qquad i=1,\dots,g$$

and

$$\sum_{i=1}^{g}\sum_{j=1}^{n}\tau_i(y_j;\Psi^{(k)})\left(\frac{\delta_j(y_j-\mu_{ij})+(1-\delta_j)(T+1)}{1+\mu_{ij}}x_{j1}\right)=0.$$

Writing in matrix form:

$$\begin{pmatrix}\frac{\partial l}{\partial \beta_{10}}\\ \vdots \\ \frac{\partial l}{\partial \beta_{g0}} \\ \frac{\partial l}{\partial \beta_1}\end{pmatrix}=\begin{pmatrix}1 & \dots & 1 & 0 & \dots & 0 & 0 & \dots & \dots & 0 & \dots & 0\\ \vdots & \dots & \vdots & \vdots & \dots & \vdots & \vdots & \dots & \dots & \vdots & \dots & \vdots\\ x_{11} & \dots & x_{n1} & x_{11} & \dots & x_{n1} & \dots & \dots & \dots & x_{11} & \dots & x_{n1}\end{pmatrix}\times$$

$$\begin{pmatrix}\tau_1(y_1;\Psi^{(k)})\left(\frac{\delta_1(y_1-\mu_{11})+(1-\delta_1)(T+1)}{1+\mu_{11}}\right)\\ \vdots \\ \tau_1(y_n;\Psi^{(k)})\left(\frac{\delta_n(y_n-\mu_{1n})+(1-\delta_n)(T+1)}{1+\mu_{1n}}\right)\\ \vdots\\ \vdots\\ \vdots\\ \tau_g(y_1;\Psi^{(k)})\left(\frac{\delta_1(y_1-\mu_{g1})+(1-\delta_1)(T+1)}{1+\mu_{g1}}\right)\\ \vdots\\ \tau_g(y_n;\Psi^{(k)})\left(\frac{\delta_n(y_n-\mu_{gn})+(1-\delta_n)(T+1)}{1+\mu_{gn}}\right)\end{pmatrix}.$$

The second derivatives of the log likelihood is given as

$$\frac{\partial^2 l}{\partial \beta_{i0}^2}=-\sum_{j=1}^{n}\tau_i(y_j;\Psi^{(k)})\frac{[\delta_j(y_j-\mu_{ij})+(1-\delta_j)(T+1)]\mu_{ij}}{(1+\mu_{ij})^2} \qquad i=1,\dots,g$$

$$\frac{\partial^2 l}{\partial \beta_{h0}\,\partial \beta_{i0}}=0 \qquad \text{for } h\neq i$$

$$\frac{\partial^2 l}{\partial \beta_1\,\partial \beta_{i0}}=-\sum_{j=1}^{n}\tau_i(y_j;\Psi^{(k)})\frac{[\delta_j(y_j-\mu_{ij})+(1-\delta_j)(T+1)]\mu_{ij}}{(1+\mu_{ij})^2}x_{j1} \qquad i=1,\dots,g$$

$$\frac{\partial^2 l}{\partial \beta_1^2}=-\sum_{i=1}^{g}\sum_{j=1}^{n}\tau_i(y_j;\Psi^{(k)})\frac{[\delta_j(y_j-\mu_{ij})+(1-\delta_j)(T+1)]\mu_{ij}}{(1+\mu_{ij})^2}x_{j1}^2.$$

Thus in matrix form

$\frac{\partial^2 l}{\partial \beta\,\partial \beta^T}=-X^TWX$ where X^T and X are given below

$$X^T=\begin{pmatrix}1 & \dots & 1 & 0 & \dots & 0 & 0 & \dots & \dots & 0 & \dots & 0\\ \vdots & \dots & \vdots & \vdots & \dots & \vdots & \vdots & \dots & \dots & \vdots & \dots & \vdots\\ x_{11} & \dots & x_{n1} & x_{11} & \dots & x_{n1} & \dots & \dots & \dots & x_{11} & \dots & x_{n1}\end{pmatrix} \text{ and } X=(X^T)^T.$$

The matrix W is given by

$$W=\begin{pmatrix} W_1 & \cdots & 0 \\ \vdots & \ddots & \vdots \\ 0 & \cdots & W_g \end{pmatrix}$$

where $W_i =$

$$\begin{pmatrix} \tau_i\left(y_1;\Psi^{(k)}\right)\frac{[\delta_1(y_1-\mu_{ij})+(1-\delta_j)(T+1)]\mu_{i1}}{(1+\mu_{i1})^2} & \cdots & 0 \\ \vdots & \ddots & \vdots \\ 0 & \cdots & \tau_i\left(y_n;\Psi^{(k)}\right)\frac{[\delta_n(y_n-\mu_{in})+(1-\delta_j)(T+1)]\mu_{in}}{(1+\mu_{in})^2} \end{pmatrix}.$$

Using Newton-Raphson method we get

$$\beta_{(r)}=\beta_{(r-1)}+[X^TWX]^{-1}X^T\begin{pmatrix} \tau_1\left(y_1;\Psi^{(k)}\right)\left(\frac{\delta_1(y_1-\mu_{11})+(1-\delta_1)(T+1)}{1+\mu_{11}}\right) \\ \vdots \\ \tau_1\left(y_n;\Psi^{(k)}\right)\left(\frac{\delta_n(y_n-\mu_{1n})+(1-\delta_n)(T+1)}{1+\mu_{1n}}\right) \\ \vdots \\ \vdots \\ \vdots \\ \tau_g\left(y_1;\Psi^{(k)}\right)\left(\frac{\delta_1(y_1-\mu_{g1})+(1-\delta_1)(T+1)}{1+\mu_{g1}}\right) \\ \vdots \\ \tau_g\left(y_n;\Psi^{(k)}\right)\left(\frac{\delta_n(y_n-\mu_{gn})+(1-\delta_n)(T+1)}{1+\mu_{gn}}\right) \end{pmatrix}.$$

which gives

$$[X^TWX]\beta_{(r)}=[X^TWX]\beta_{(r-1)}+X^T\begin{pmatrix} \tau_1\left(y_1;\Psi^{(k)}\right)\left(\frac{\delta_1(y_1-\mu_{11})+(1-\delta_1)(T+1)}{1+\mu_{11}}\right) \\ \vdots \\ \tau_1\left(y_n;\Psi^{(k)}\right)\left(\frac{\delta_n(y_n-\mu_{1n})+(1-\delta_n)(T+1)}{1+\mu_{1n}}\right) \\ \vdots \\ \vdots \\ \vdots \\ \tau_g\left(y_1;\Psi^{(k)}\right)\left(\frac{\delta_1\left(y_1-\mu_{g1}\right)+(1-\delta_1)(T+1)}{1+\mu_{g1}}\right) \\ \vdots \\ \tau_g\left(y_n;\Psi^{(k)}\right)\left(\frac{\delta_n\left(y_n-\mu_{gn}\right)+(1-\delta_n)(T+1)}{1+\mu_{gn}}\right) \end{pmatrix}$$

Using the relation $X\beta_{(r-1)}=\eta_{(r-1)}$we get

$$[X^TWX]\beta_{(r)} = X^TW\eta_{(r-1)} + X^T\begin{pmatrix} \tau_1\left(y_1;\Psi^{(k)}\right)\left(\frac{\delta_1(y_1-\mu_{11})+(1-\delta_1)(T+1)}{1+\mu_{11}}\right) \\ \vdots \\ \tau_1\left(y_n;\Psi^{(k)}\right)\left(\frac{\delta_n(y_n-\mu_{1n})+(1-\delta_n)(T+1)}{1+\mu_{1n}}\right) \\ \vdots \\ \vdots \\ \vdots \\ \tau_g\left(y_1;\Psi^{(k)}\right)\left(\frac{\delta_1(y_1-\mu_{g1})+(1-\delta_1)(T+1)}{1+\mu_{g1}}\right) \\ \vdots \\ \tau_g\left(y_n;\Psi^{(k)}\right)\left(\frac{\delta_n(y_n-\mu_{gn})+(1-\delta_n)(T+1)}{1+\mu_{gn}}\right) \end{pmatrix}.$$

$$= X^TW\left(\eta_{(r-1)} + W^{-1}\begin{pmatrix} \tau_1\left(y_1;\Psi^{(k)}\right)\left(\frac{\delta_1(y_1-\mu_{11})+(1-\delta_1)(T+1)}{1+\mu_{11}}\right) \\ \vdots \\ \tau_1\left(y_n;\Psi^{(k)}\right)\left(\frac{\delta_n(y_n-\mu_{1n})+(1-\delta_n)(T+1)}{1+\mu_{1n}}\right) \\ \vdots \\ \vdots \\ \vdots \\ \tau_g\left(y_1;\Psi^{(k)}\right)\left(\frac{\delta_1(y_1-\mu_{g1})+(1-\delta_1)(T+1)}{1+\mu_{g1}}\right) \\ \vdots \\ \tau_g\left(y_n;\Psi^{(k)}\right)\left(\frac{\delta_n(y_n-\mu_{gn})+(1-\delta_n)(T+1)}{1+\mu_{gn}}\right) \end{pmatrix}\right)$$

$$= X^TWz$$

where $z = \left(\eta_{(r-1)} + W^{-1}\begin{pmatrix} \tau_1\left(y_1;\Psi^{(k)}\right)\left(\frac{\delta_1(y_1-\mu_{11})+(1-\delta_1)(T+1)}{1+\mu_{11}}\right) \\ \vdots \\ \tau_1\left(y_n;\Psi^{(k)}\right)\left(\frac{\delta_n(y_n-\mu_{1n})+(1-\delta_n)(T+1)}{1+\mu_{1n}}\right) \\ \vdots \\ \vdots \\ \vdots \\ \tau_g\left(y_1;\Psi^{(k)}\right)\left(\frac{\delta_1(y_1-\mu_{g1})+(1-\delta_1)(T+1)}{1+\mu_{g1}}\right) \\ \vdots \\ \tau_g\left(y_n;\Psi^{(k)}\right)\left(\frac{\delta_n(y_n-\mu_{gn})+(1-\delta_n)(T+1)}{1+\mu_{gn}}\right) \end{pmatrix}\right)$ and thus

$$\beta_{(r)} = [X^TWX]^{-1}X^TWz.$$
