# Supplementary material for: A distance-type measure approach to the analysis of copy number variation in DNA sequencing data
Source: BMC Genomics. 2019 Apr 4;20(Suppl 2):195. doi: 10.1186/s12864-019-5491-x (PMC6456939; doi:10.1186/s12864-019-5491-x)
Supplement: Supplementary file 2 — Supplemental figures. (PDF 1697 kb) [file 12864_2019_5491_MOESM2_ESM.pdf]

Figure legends

Supplemental Figure 1: Bias of the parameter estimates ($\pi_1 = 0.3, \pi_2 = 0.5, \pi_3 = 0.2$ and $p_1 = 0.002, p_2 = 0.02, p_3 = 0.2$).

Supplemental Figure 2: Standard deviation (Stdev) of the parameter estimates ($\pi_1 = 0.3, \pi_2 = 0.5, \pi_3 = 0.2$ and $p_1 = 0.002, p_2 = 0.02, p_3 = 0.2$).

Supplemental Figure 3: RMSE of the parameter estimates ($\pi_1 = 0.3, \pi_2 = 0.5, \pi_3 = 0.2$ and $p_1 = 0.002, p_2 = 0.02, p_3 = 0.2$).

Supplemental Figure 4: Box plots of estimates of π_1, π_2, π_3 from simulations ($\pi_1 = 0.3, \pi_2 = 0.5, \pi_3 = 0.2$ and $p_1 = 0.002, p_2 = 0.02, p_3 = 0.2$). 1=(N=100,T=250); 2= (N=100, T=500); 3=(N=100, T=1000); 4=(N=100, no censoring); 5=(N=250, T=250); 6=(N=250, T=500); 7=(N=250, T=1000); 8=(N=250, no censoring); 9=(N=500, T=250); 10=(N=500, T=500); 11=(N=500,T=1000); 12=(N=500, no censoring); 13=(N=1000, T=250); 14=(N=1000, T=500); 15=(N=1000, T=1000); 16=(N=1000, no censoring); 17=(N=5000, T=250); 18=(N=5000, T=500); 19=(N=5000, T=1000);20=(N=5000, no censoring).

Supplemental Figure 5: Box plots of estimates of p_1, p, p_3 from simulations ($\pi_1 = 0.3, \pi_2 = 0.5, \pi_3 = 0.2$ and $p_1 = 0.002, p_2 = 0.02, p_3 = 0.2$). 1=(N=100,T=250); 2= (N=100, T=500); 3=(N=100, T=1000); 4=(N=100, no censoring); 5=(N=250, T=250); 6=(N=250, T=500); 7=(N=250, T=1000); 8=(N=250, no censoring); 9=(N=500, T=250); 10=(N=500, T=500); 11=(N=500,T=1000); 12=(N=500, no censoring); 13=(N=1000, T=250); 14=(N=1000, T=500); 15=(N=1000, T=1000); 16=(N=1000, no censoring); 17=(N=5000, T=250); 18=(N=5000, T=500); 19=(N=5000, T=1000);20=(N=5000, no censoring).

Supplemental Figure 6: Bias of the parameter estimates ($\pi_1 = 0.008,\ \pi_2 = 0.754,\ \pi_3 = 0.238$ and $p_1 = 0.999,\ p_2 = 0.011,\ p_3 = 0.0006$).

Supplemental Figure 7: Standard deviation (Stdev) of the parameter estimates ($\pi_1 = 0.008,\ \pi_2 = 0.754,\ \pi_3 = 0.238$ and $p_1 = 0.999,\ p_2 = 0.011,\ p_3 = 0.0006$).

Supplemental Figure 8: RMSE of the parameter estimates ($\pi_1 = 0.008,\ \pi_2 = 0.754,\ \pi_3 = 0.238$ and $p_1 = 0.999,\ p_2 = 0.011,\ p_3 = 0.0006$).

Supplemental Figure 9: Box plots of estimates of π_1, π_2, π_3 from simulations ($\pi_1 = 0.008,\ \pi_2 = 0.754,\ \pi_3 = 0.238$ and $p_1 = 0.999,\ p_2 = 0.011,\ p_3 = 0.0006$). 1=(N=100,T=1000); 2=(N=100, T=2500); 3=(N=100, T=5000); 4=(N=100, no censoring); 5=(N=250, T=1000); 6=(N=250, T=2500); 7=(N=250, T=5000); 8=(N=250, no censoring); 9=(N=500, T=1000); 10=(N=500, T=2500); 11=(N=500,T=5000); 12=(N=500, no censoring); 13=(N=1000, T=1000); 14=(N=1000, T=2500); 15=(N=1000, T=5000); 16=(N=1000, no censoring); 17=(N=5000, T=1000); 18=(N=5000, T=2500); 19=(N=5000, T=5000);20=(N=5000, no censoring).

Supplemental Figure 10: Box plots of estimates of p_1, p, p_3 from simulations ($\pi_1 = 0.008,\ \pi_2 = 0.754,\ \pi_3 = 0.238$ and $p_1 = 0.999,\ p_2 = 0.011,\ p_3 = 0.0006$). 1=(N=100,T=1000); 2=(N=100, T=2500); 3=(N=100, T=5000); 4=(N=100, no censoring); 5=(N=250, T=1000); 6=(N=250, T=2500); 7=(N=250, T=5000); 8=(N=250, no censoring); 9=(N=500, T=1000); 10=(N=500, T=2500); 11=(N=500,T=5000); 12=(N=500, no censoring); 13=(N=1000, T=1000); 14=(N=1000, T=2500); 15=(N=1000, T=5000); 16=(N=1000, no censoring); 17=(N=5000, T=1000); 18=(N=5000, T=2500); 19=(N=5000, T=5000);20=(N=5000, no censoring).

Supplemental Figure 11: Box plots of estimates of π_1, π_2, π_3 from simulations. 1=(N=10000,T=570); 2= (N=10000, T=937); 3=(N=10000, T=1309); 4=(N=10000, no censoring).

Supplemental Figure 12: Box plots of estimates of $\beta_{10}, \beta_{20}, \beta_{30}, \beta_1$ from simulations. 1=(N=10000,T=570); 2= (N=10000, T=937); 3=(N=10000, T=1309); 4=(N=10000, no censoring). Figure legends

Supplemental Figure 13: Box plots of estimates of π_1, π_2, π_3 from simulations. 1= (N=10000, T=549); 2= (N=10000, T=895); 3= (N=10000, T=1241); 4= (N=10000, no censoring).

Supplemental Figure 14: Box plots of estimates of $\beta_{10}, \beta_{20}, \beta_{30}, \beta_1$ from simulations. 1= (N=10000, T=549); 2= (N=10000, T=895); 3= (N=10000, T=1241); 4= (N=10000, no censoring).

Supplemental Figure 15: ECDF of p-values versus the CDF of a Uniform (0,1) (200 Bootstrap samples).

Supplemental Figure 16: ECDF of p-values versus the CDF of a Uniform (0,1) (100 Bootstrap samples).

Supplemental Figure 17: Q-Q plots (INT) for randomly selected regions of length 100,000 bps along the positions 200,000-38,000,000 bps on chromosome 9 (normal sample).

Supplemental Figure 18: Q-Q plots (INT) for randomly selected regions of length 100,000 bps along the positions 200,000-38,000,000 bps on chromosome 9 (tumor sample).

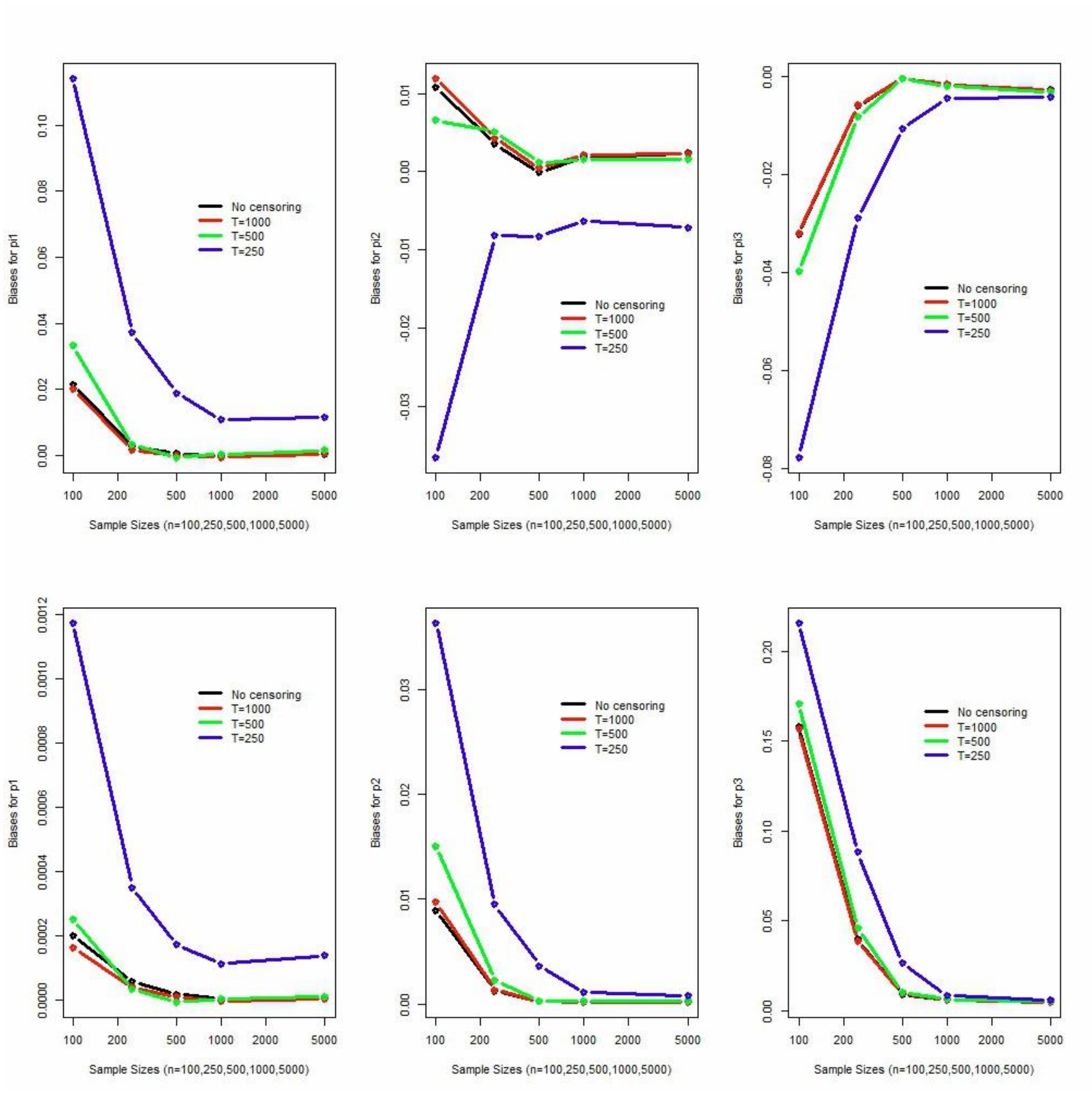

Supplemental Figure 1: Bias of the parameter estimates ($\pi_1 = 0.3, \pi_2 = 0.5, \pi_3 = 0.2$ and $p_1 = 0.002, p_2 = 0.02, p_3 = 0.2$).

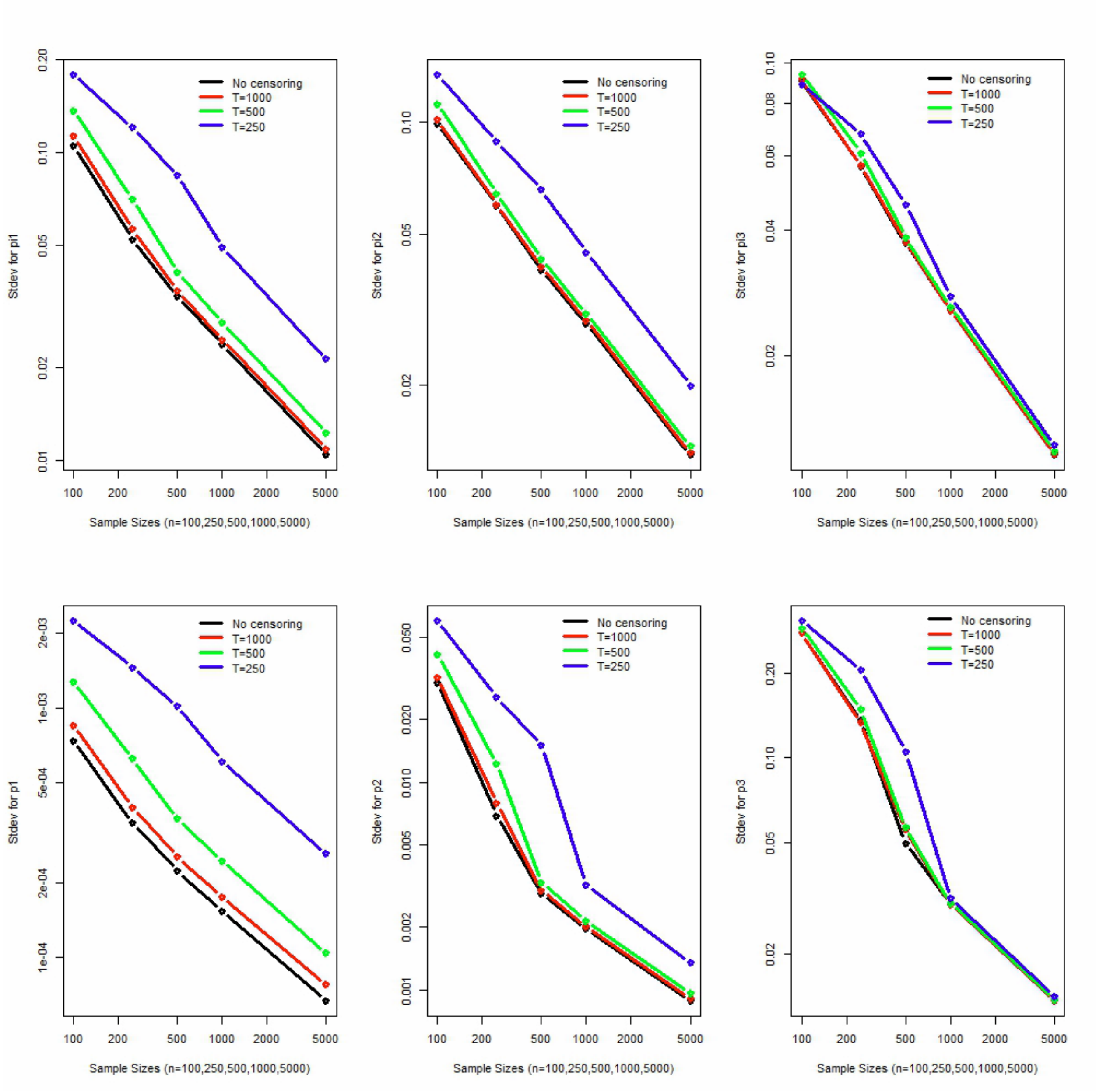

Supplemental Figure 2: Standard deviation (Stdev) of the parameter estimates ($\pi_1 = 0.3, \pi_2 = 0.5, \pi_3 = 0.2$ and $p_1 = 0.002, p_2 = 0.02, p_3 = 0.2$).

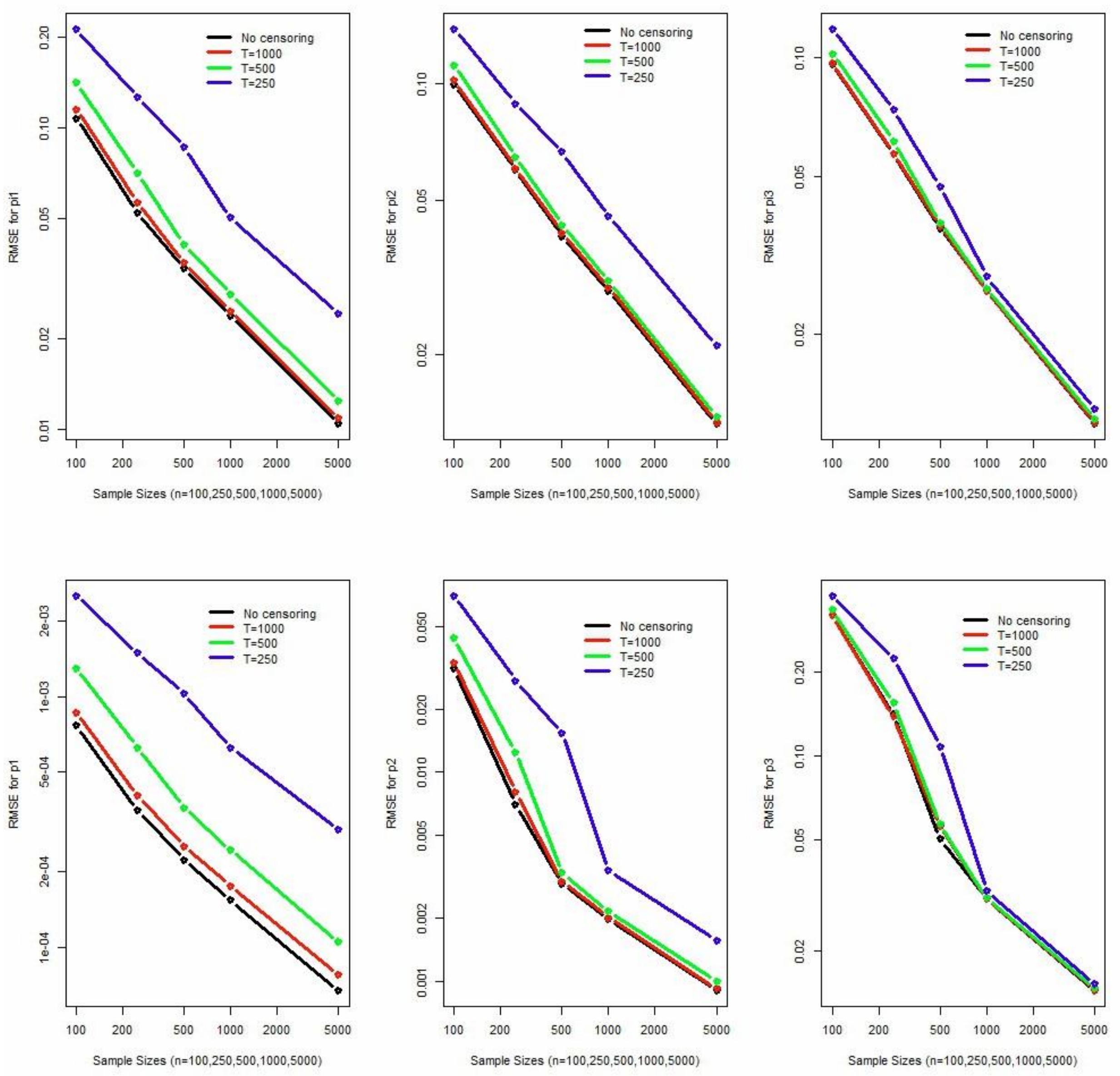

Supplemental Figure 3: RMSE of the parameter estimates ($\pi_1 = 0.3, \pi_2 = 0.5, \pi_3 = 0.2$ and $p_1 = 0.002, p_2 = 0.02, p_3 = 0.2$).

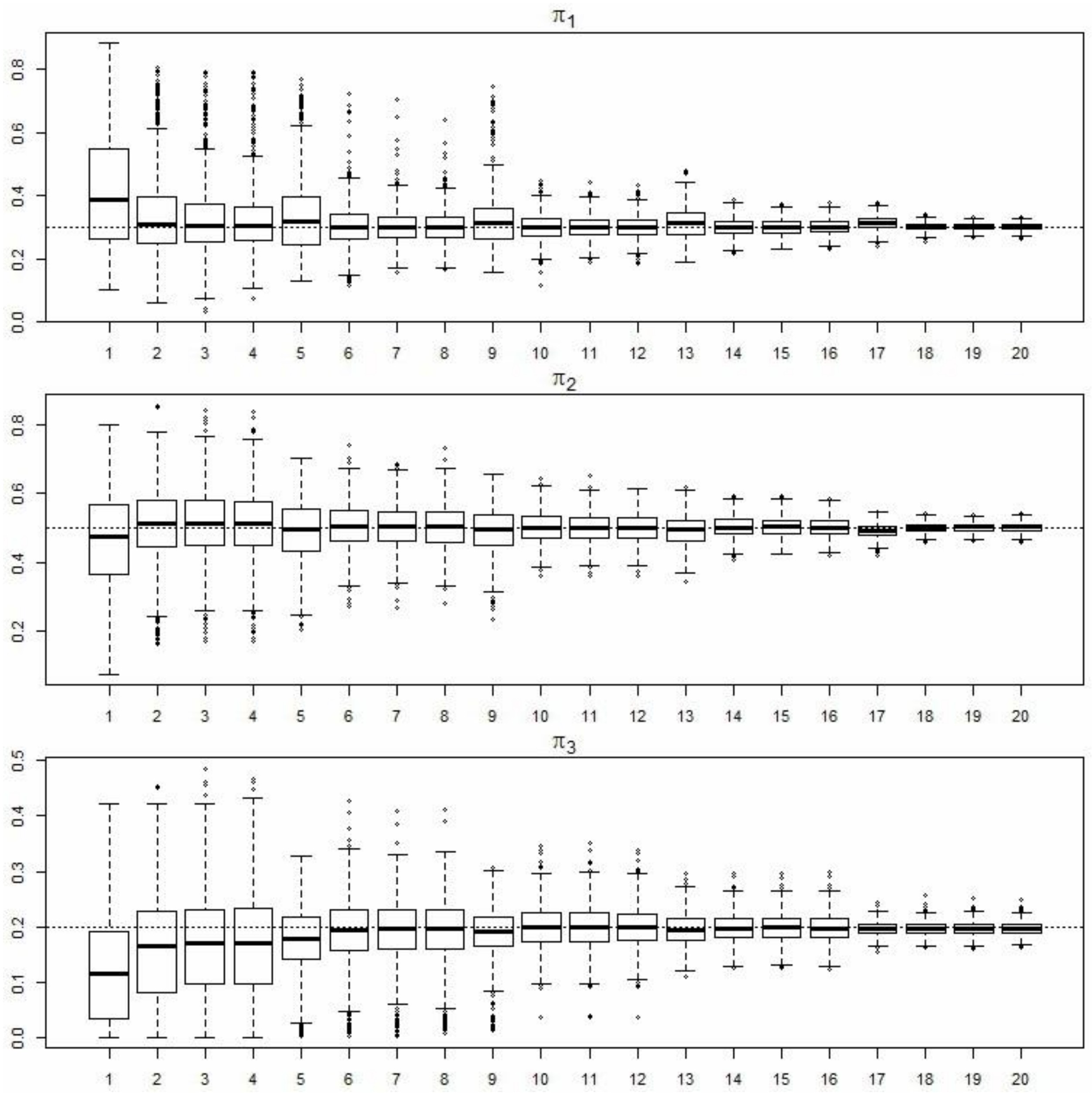

Supplemental Figure 4: Box plots of estimates of π_1, π_2, π_3 from simulations ($\pi_1 = 0.3, \pi_2 = 0.5, \pi_3 = 0.2$ and $p_1 = 0.002, p_2 = 0.02, p_3 = 0.2$). 1=(N=100,T=250); 2= (N=100, T=500); 3=(N=100, T=1000); 4=(N=100, no censoring); 5=(N=250, T=250); 6=(N=250, T=500); 7=(N=250, T=1000); 8=(N=250, no censoring); 9=(N=500, T=250); 10=(N=500, T=500); 11=(N=500,T=1000); 12=(N=500, no censoring); 13=(N=1000, T=250); 14=(N=1000, T=500); 15=(N=1000, T=1000); 16=(N=1000, no censoring); 17=(N=5000, T=250); 18=(N=5000, T=500); 19=(N=5000, T=1000);20=(N=5000, no censoring).

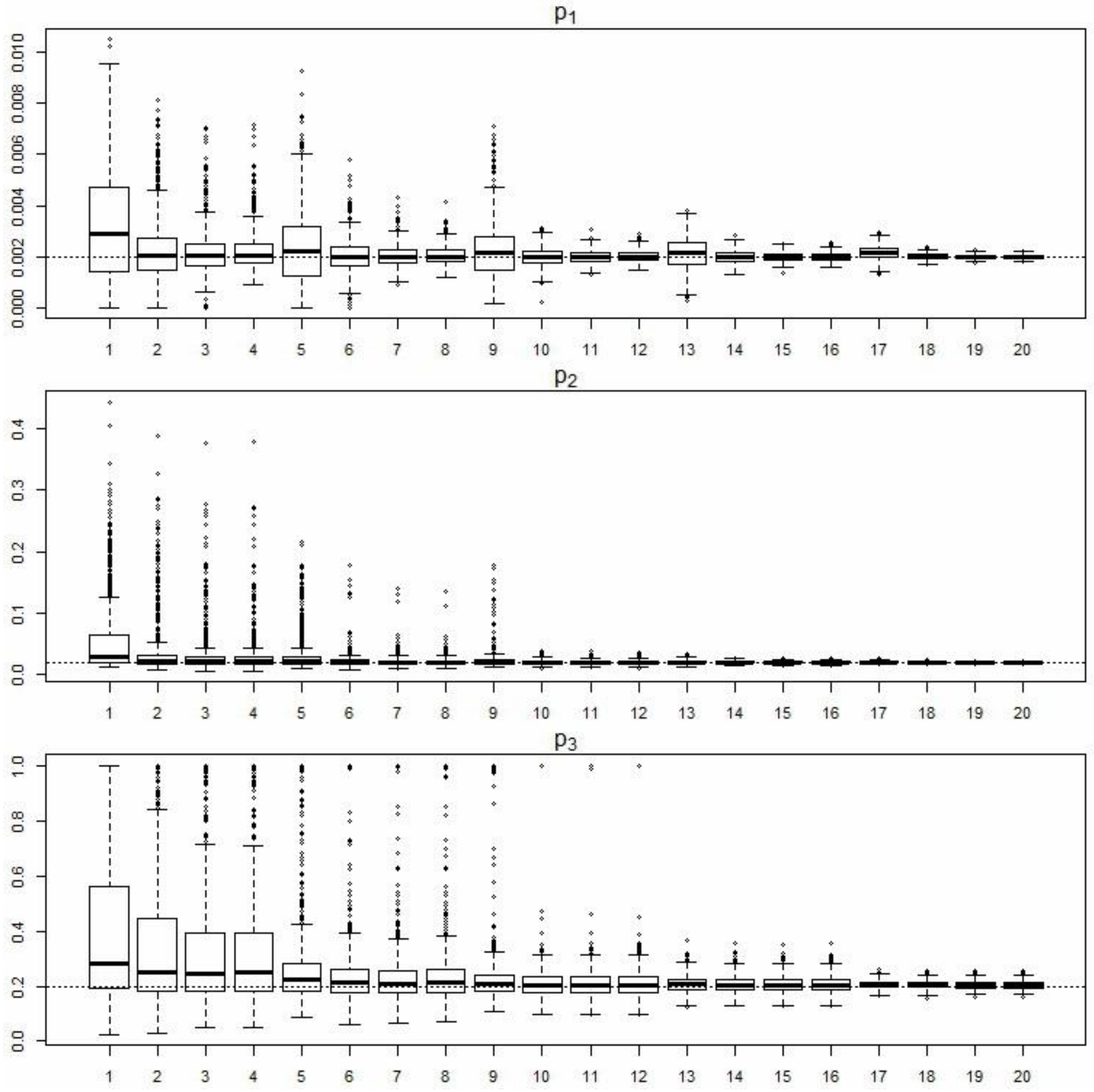

Supplemental Figure 5: Box plots of estimates of p_1, p, p_3 from simulations ($\pi_1 = 0.3, \pi_2 = 0.5, \pi_3 = 0.2$ and $p_1 = 0.002, p_2 = 0.02, p_3 = 0.2$). 1=(N=100,T=250); 2= (N=100, T=500); 3=(N=100, T=1000); 4=(N=100, no censoring); 5=(N=250, T=250); 6=(N=250, T=500); 7=(N=250, T=1000); 8=(N=250, no censoring); 9=(N=500, T=250); 10=(N=500, T=500); 11=(N=500,T=1000); 12=(N=500, no censoring); 13=(N=1000, T=250); 14=(N=1000, T=500); 15=(N=1000, T=1000); 16=(N=1000, no censoring); 17=(N=5000, T=250); 18=(N=5000, T=500); 19=(N=5000, T=1000);20=(N=5000, no censoring).

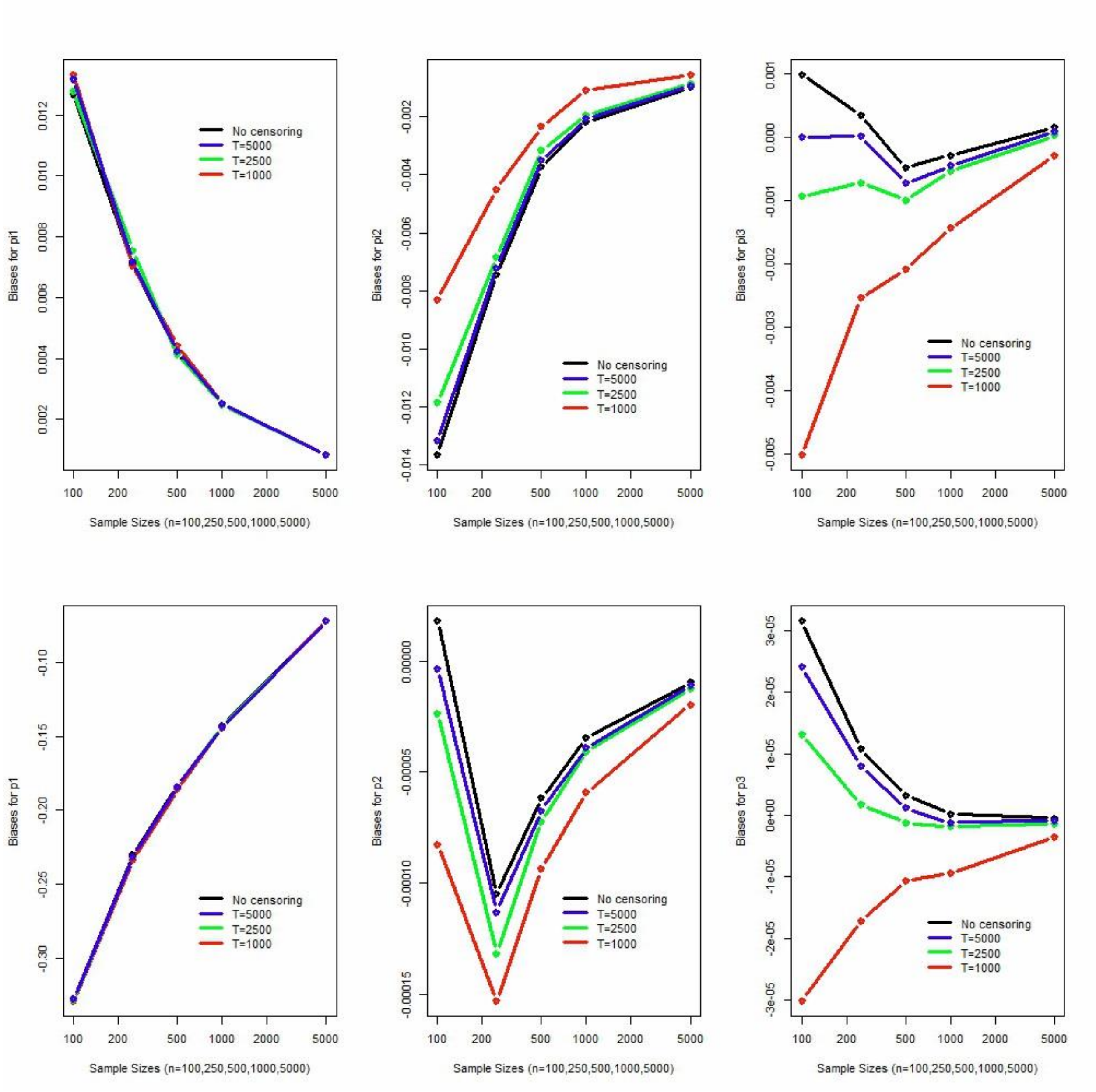

Supplemental Figure 6: Bias of the parameter estimates ($\pi_1 = 0.008,\ \pi_2 = 0.754,\ \pi_3 = 0.238$ and $p_1 = 0.999,\ p_2 = 0.011,\ p_3 = 0.0006$).

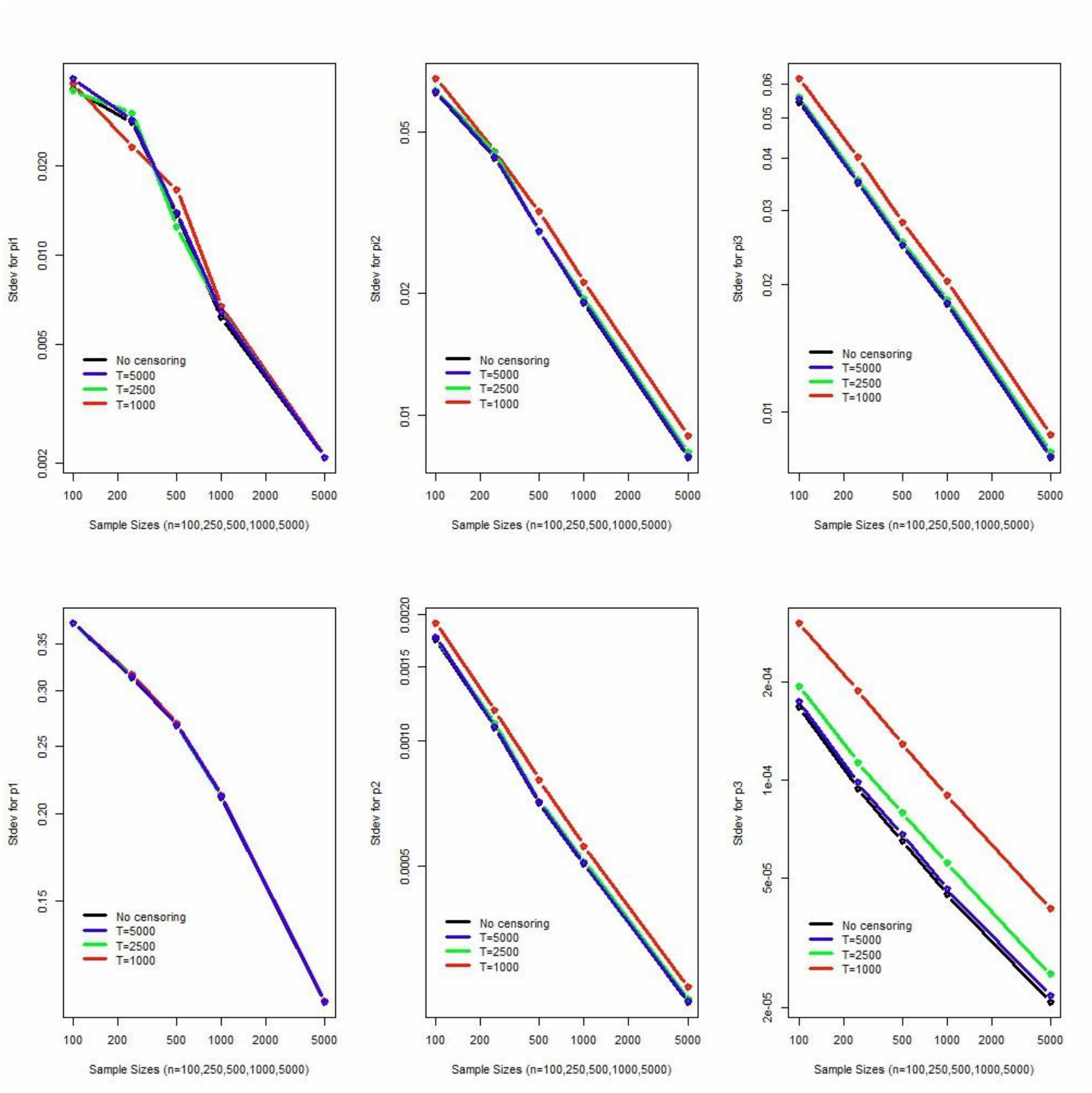

Supplemental Figure 7: Standard deviation (Stdev) of the parameter estimates ($\pi_1 = 0.008$, $\pi_2 = 0.754$, $\pi_3 = 0.238$ and $p_1 = 0.999$, $p_2 = 0.011$, $p_3 = 0.0006$).

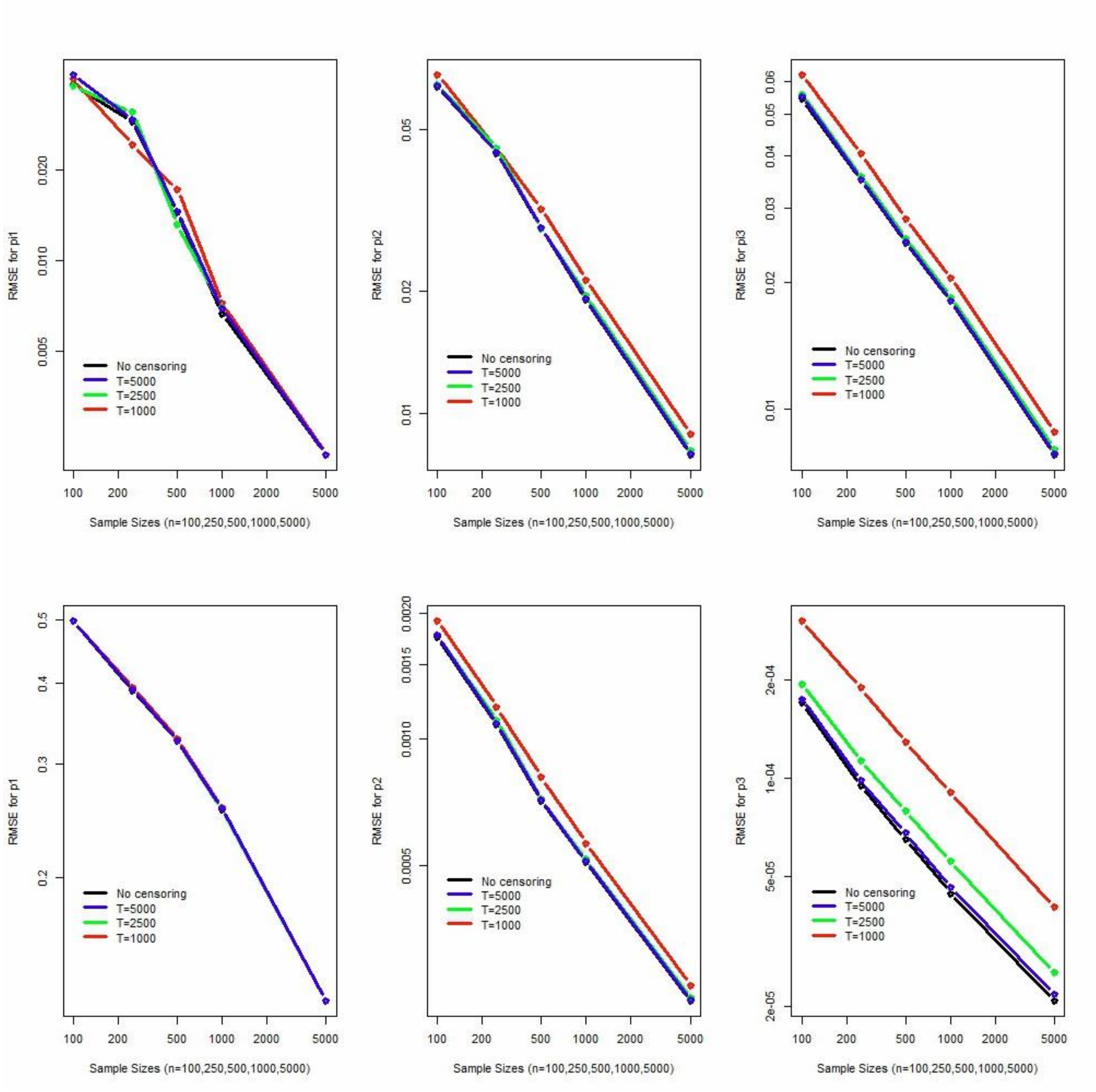

Supplemental Figure 8: RMSE of the parameter estimates ($\pi_1 = 0.008,\ \pi_2 = 0.754,\ \pi_3 = 0.238$ and $p_1 = 0.999,\ p_2 = 0.011,\ p_3 = 0.0006$).

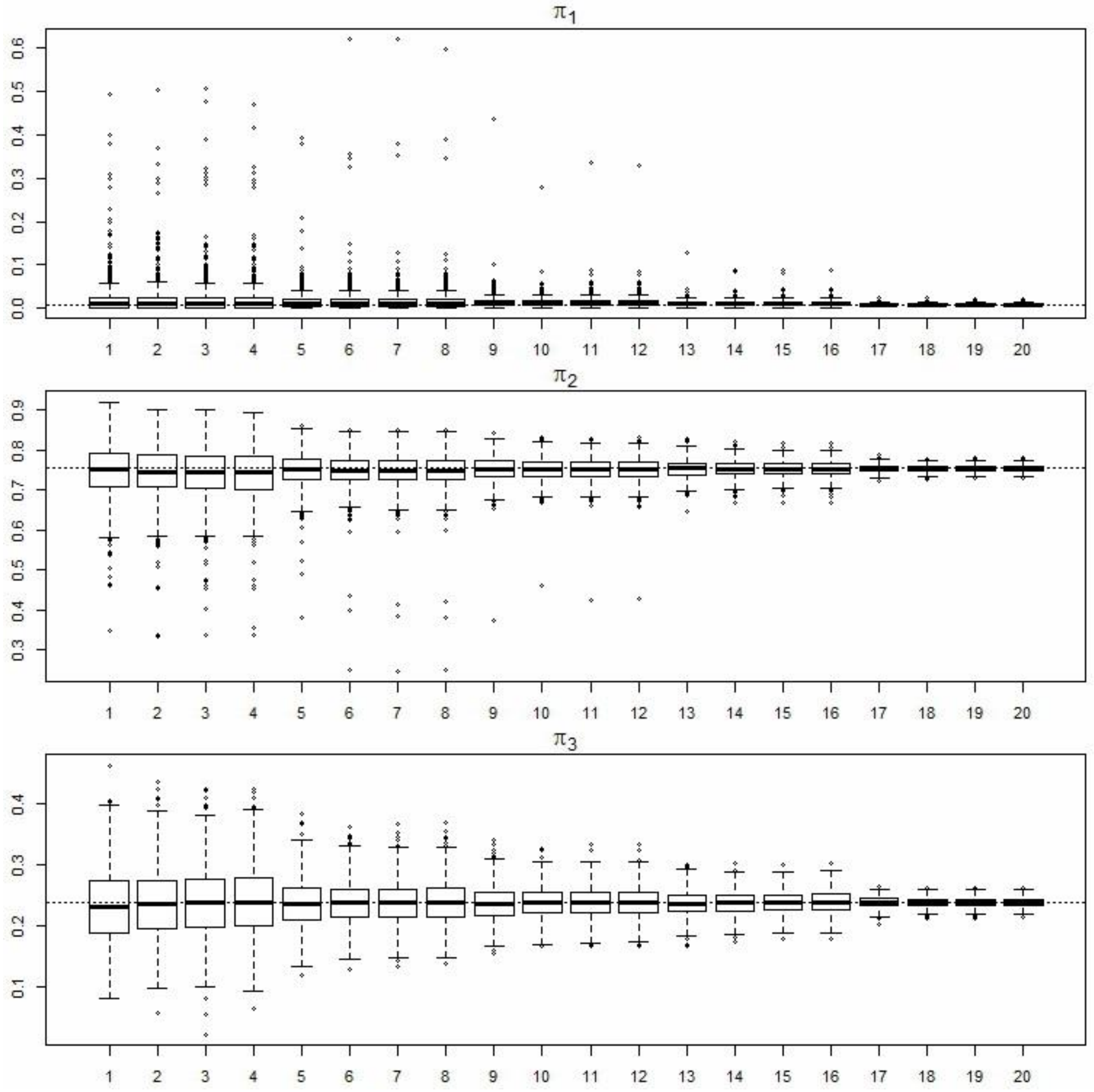

Supplemental Figure 9: Box plots of estimates of π_1, π_2, π_3 from simulations ($\pi_1 = 0.008$, $\pi_2 = 0.754$, $\pi_3 = 0.238$ and $p_1 = 0.999$, $p_2 = 0.011$, $p_3 = 0.0006$). 1=(N=100,T=1000); 2=(N=100, T=2500); 3=(N=100, T=5000); 4=(N=100, no censoring); 5=(N=250, T=1000); 6=(N=250, T=2500); 7=(N=250, T=5000); 8=(N=250, no censoring); 9=(N=500, T=1000); 10=(N=500, T=2500); 11=(N=500,T=5000); 12=(N=500, no censoring); 13=(N=1000, T=1000); 14=(N=1000, T=2500); 15=(N=1000, T=5000); 16=(N=1000, no censoring); 17=(N=5000, T=1000); 18=(N=5000, T=2500); 19=(N=5000, T=5000);20=(N=5000, no censoring).

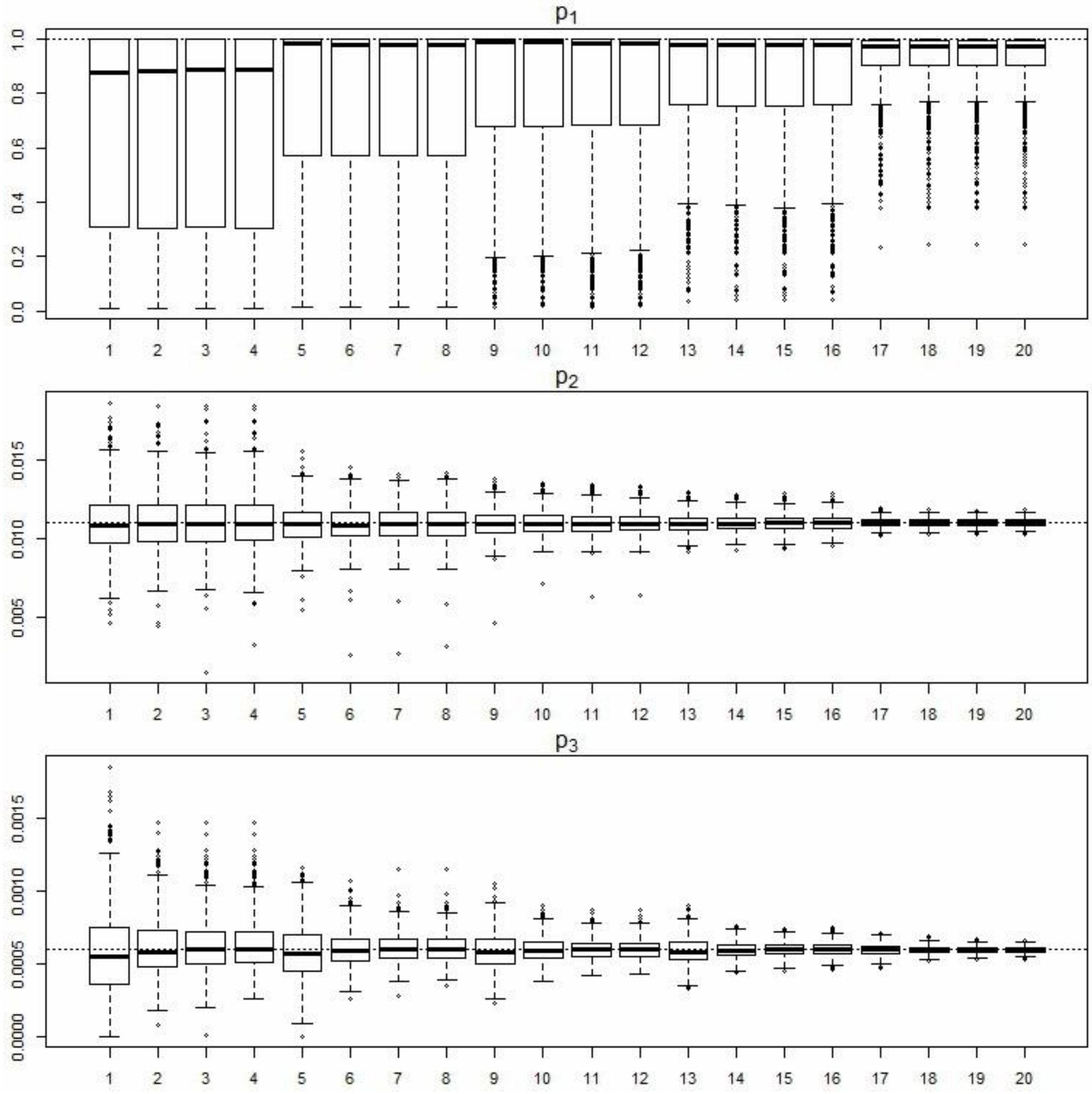

Supplemental Figure 10: Box plots of estimates of p_1, p, p_3 from simulations ($\pi_1 = 0.008,\ \pi_2 = 0.754,\ \pi_3 = 0.238$ and $p_1 = 0.999,\ p_2 = 0.011,\ p_3 = 0.0006$). 1=(N=100,T=1000); 2=(N=100, T=2500); 3=(N=100, T=5000); 4=(N=100, no censoring); 5=(N=250, T=1000); 6=(N=250, T=2500); 7=(N=250, T=5000); 8=(N=250, no censoring); 9=(N=500, T=1000); 10=(N=500, T=2500); 11=(N=500,T=5000); 12=(N=500, no censoring); 13=(N=1000, T=1000); 14=(N=1000, T=2500); 15=(N=1000, T=5000); 16=(N=1000, no censoring); 17=(N=5000, T=1000); 18=(N=5000, T=2500); 19=(N=5000, T=5000);20=(N=5000, no censoring).

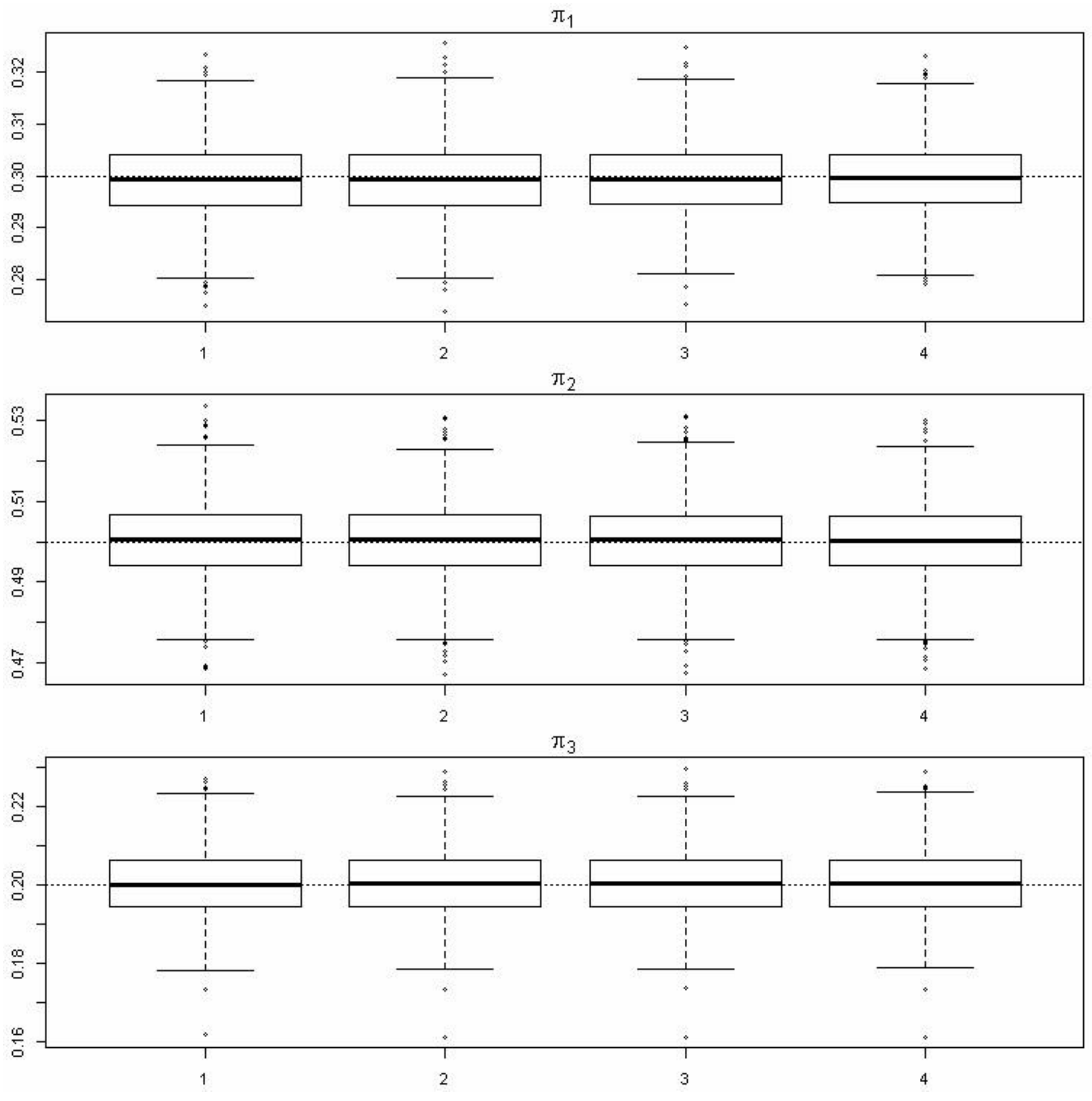

Supplemental Figure 11: Box plots of estimates of π_1, π_2, π_3 from simulations. 1=(N=10000,T=570); 2= (N=10000, T=937); 3=(N=10000, T=1309); 4=(N=10000, no censoring).

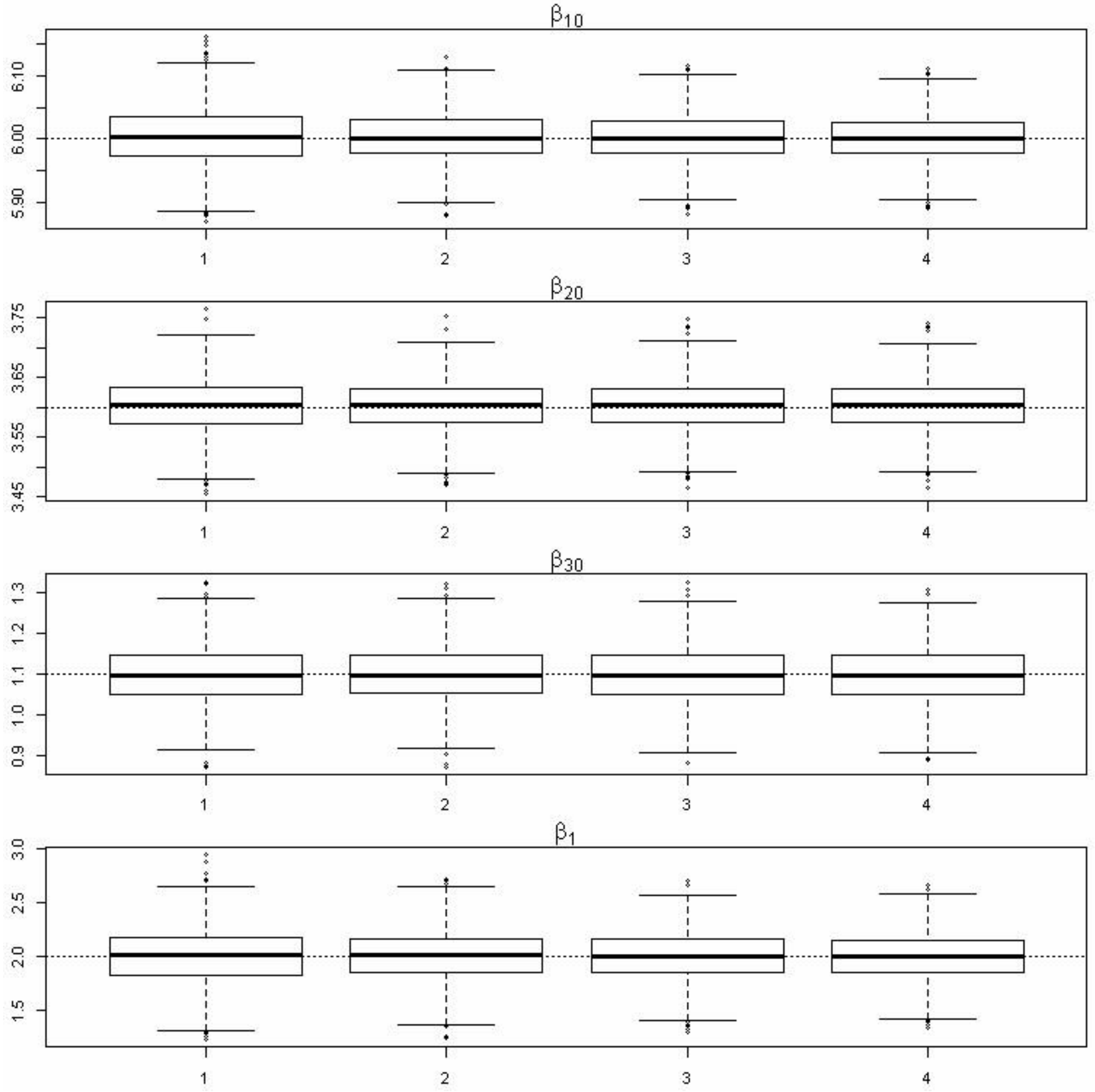

Supplemental Figure 12: Box plots of estimates of $\beta_{10}, \beta_{20}, \beta_{30}, \beta_1$ from simulations. 1=(N=10000,T=570); 2= (N=10000, T=937); 3=(N=10000, T=1309); 4=(N=10000, no censoring). Figure legends

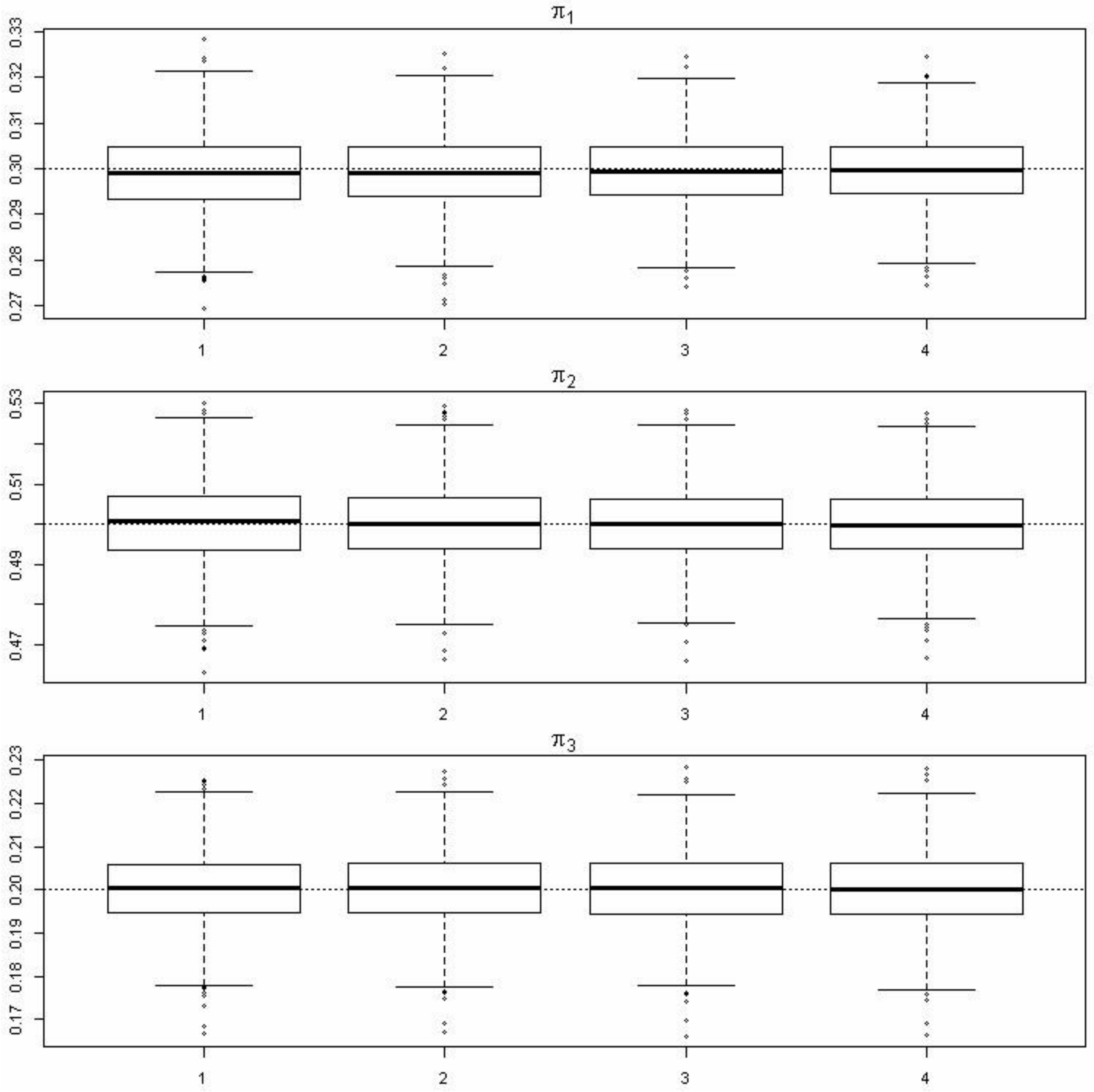

Supplemental Figure 13: Box plots of estimates of π_1, π_2, π_3 from simulations. 1= (N=10000, T=549); 2= (N=10000, T=895); 3= (N=10000, T=1241); 4= (N=10000, no censoring).

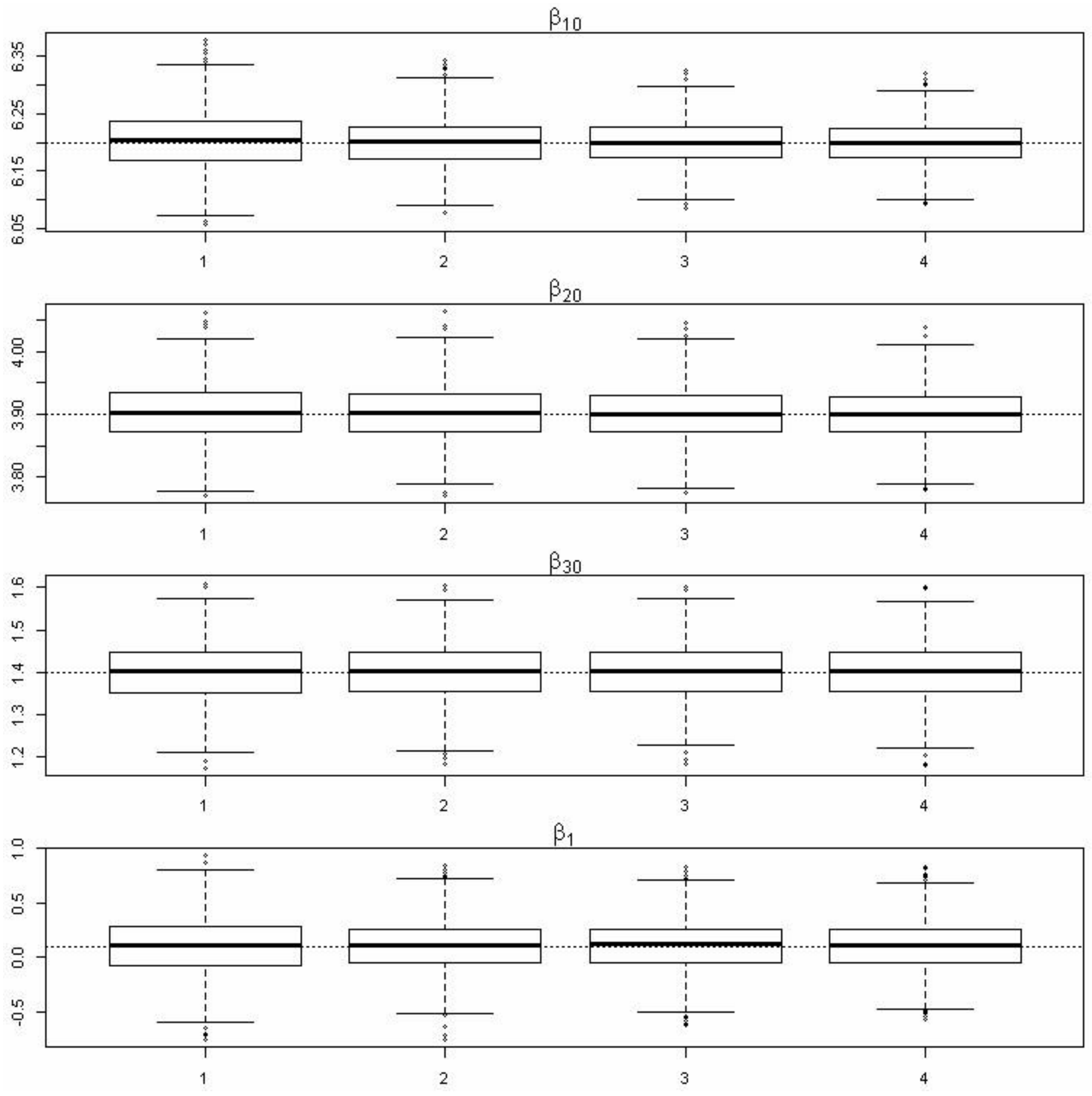

Supplemental Figure 14: Box plots of estimates of $\beta_{10}, \beta_{20}, \beta_{30}, \beta_1$ from simulations. 1= (N=10000, T=549); 2= (N=10000, T=895); 3= (N=10000, T=1241); 4= (N=10000, no censoring).

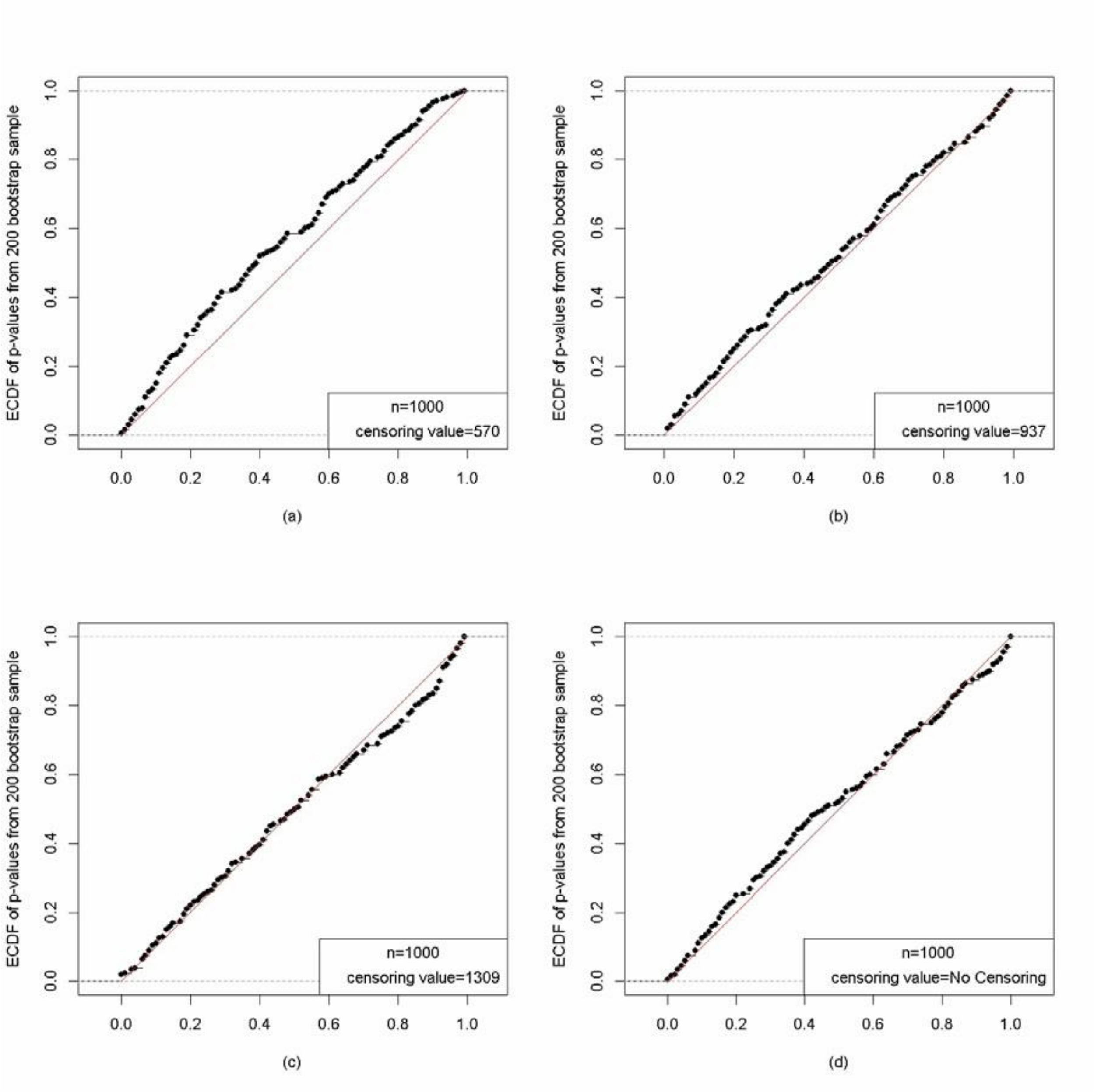

Supplemental Figure 15: ECDF of p-values versus the CDF of a Uniform (0,1) (200 Bootstrap samples).

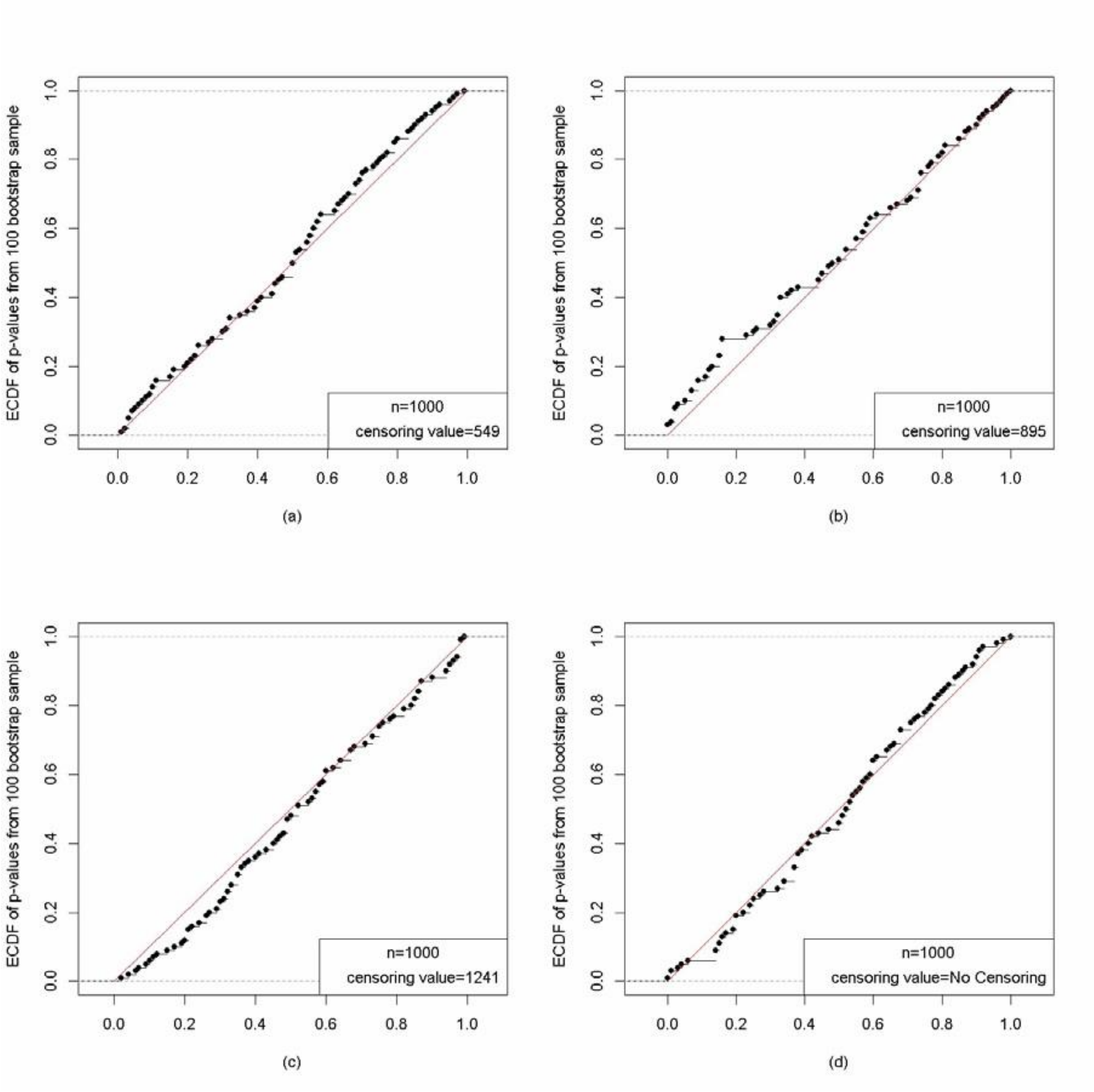

Supplemental Figure 16: ECDF of p-values versus the CDF of a Uniform (0,1) (100 Bootstrap samples).

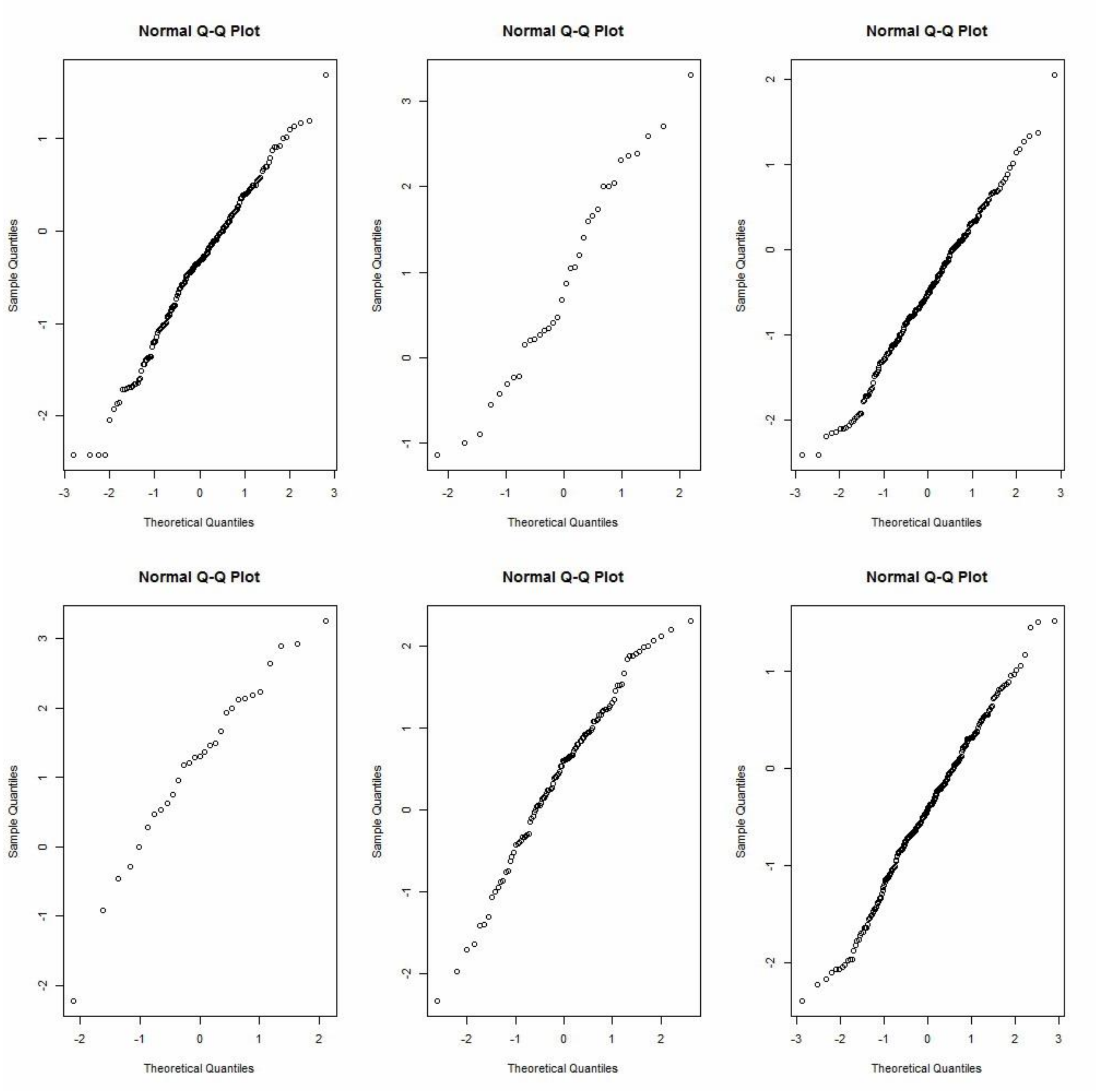

Supplemental Figure 17: Q-Q plots (INT) for randomly selected regions of length 100,000 bps along the positions 200,000-38,000,000 bps on chromosome 9 (normal sample).

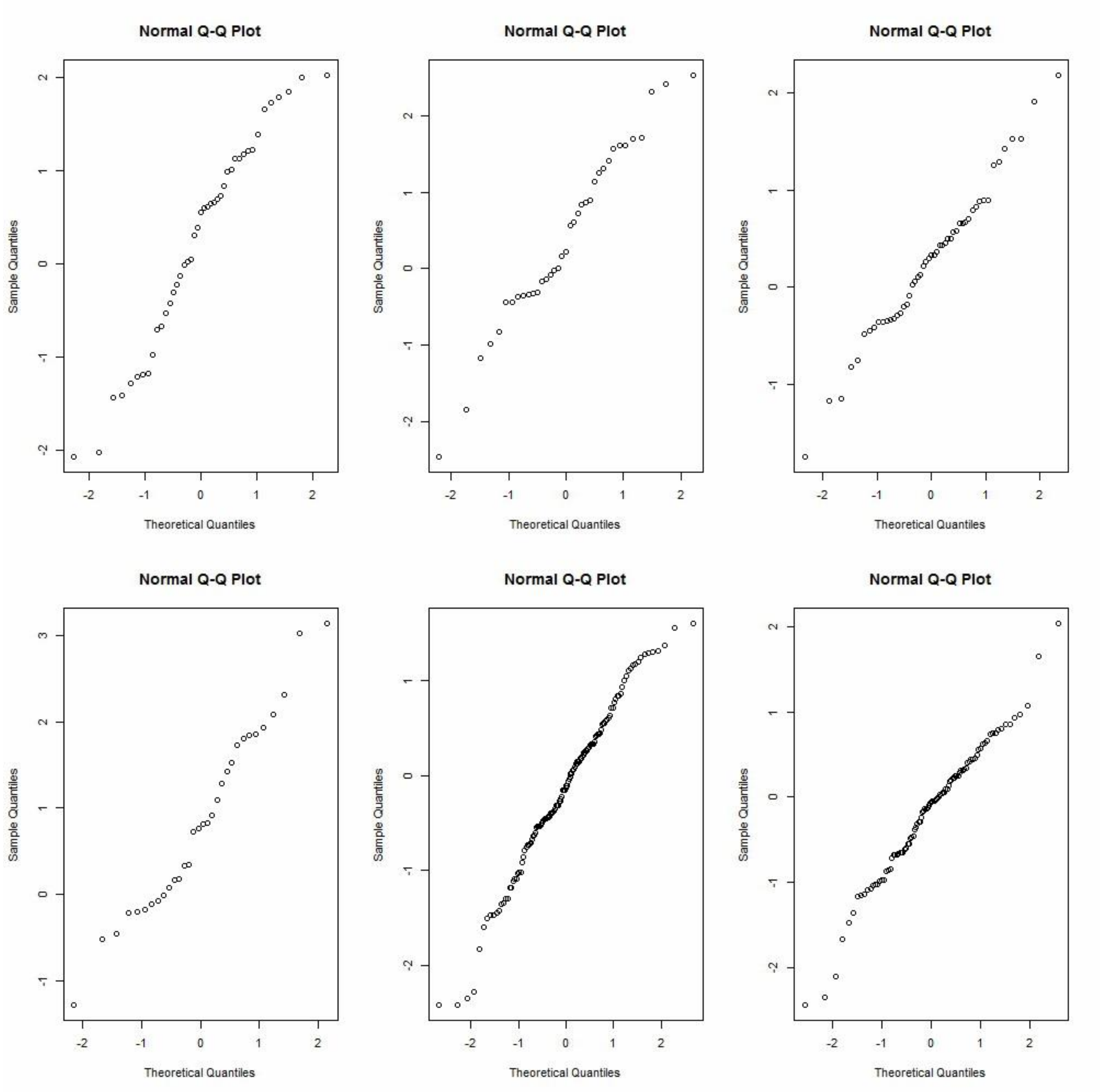

Supplemental Figure 18: Q-Q plots (INT) for randomly selected regions of length 100,000 bps along the positions 200,000-38,000,000 bps on chromosome 9 (tumor sample).
